# Supplementary material for: High-throughput identification of pathogen effector proteins that target host transcription by dual perturb-seq
Source: Cell Host Microbe. Author manuscript; Available in PMC 2026 Apr 9. (PMC12033024; doi:10.1016/j.chom.2023.09.003)
Supplement: Suppl files [file EMS212870-supplement-Suppl_files.zip › 1-s2.0-S1931312823003700-mmc1.pdf]

Cell Host & Microbe, Volume *31*

Supplemental information

High-throughput identification of *Toxoplasma gondii* effector proteins that target host cell transcription

Simon Butterworth, Kristina Kordova, Sambamurthy Chandrasekaran, Kaitlin K. Thomas, Francesca Torelli, Eloise J. Lockyer, Amelia Edwards, Robert Goldstone, Anita A. Koshy, and Moritz Treeck

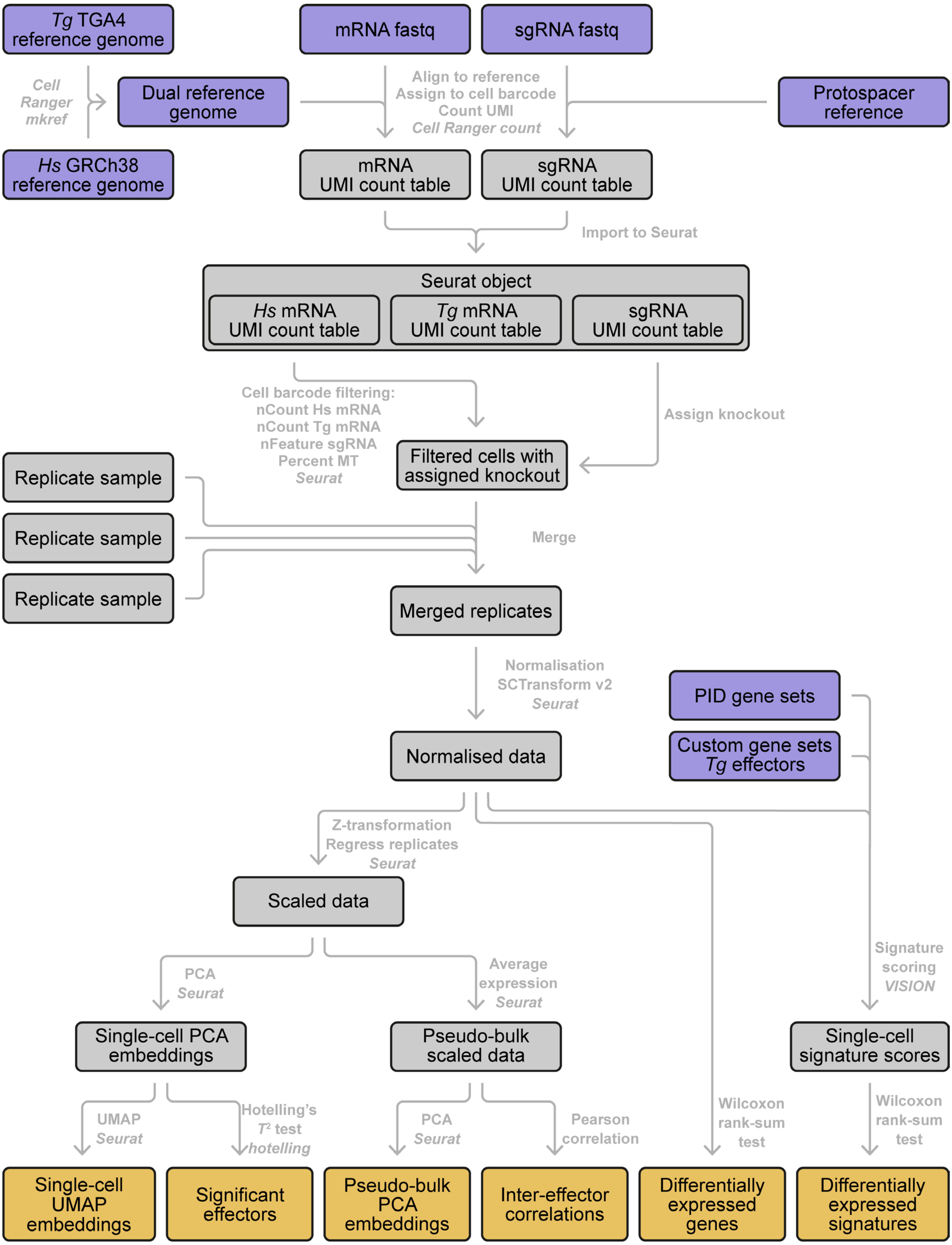

Figure S1. Schematic of dual perturb-seq data processing and analysis, related to STAR Methods.

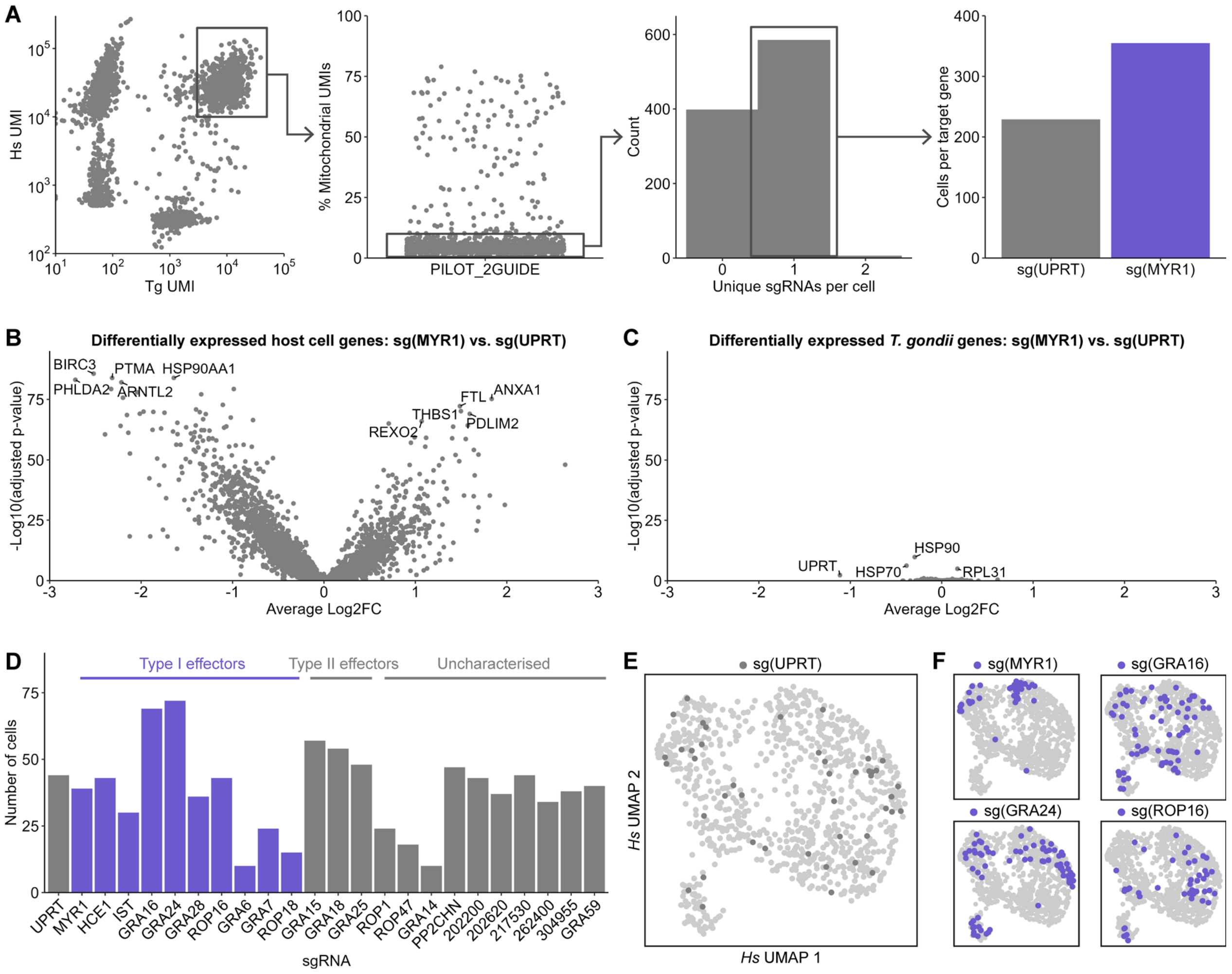

Figure S2. Filtering and assignment of sgRNA identity to dual perturb-seq transcriptomes, related to Figure 1.

A. Typical cell barcode filtering strategy for dual perturb-seq data. Cell barcodes are filtered to retain only those with high counts of both host cell and *T. gondii* UMIs, a low percentage of host cell UMIs deriving from mitochondrial transcripts, and in which only a single sgRNA species is detected.

B. Differentially expressed host cell genes for sg(MYR1)-expressing cells compared to sg(UPRT)-expressing cells in the 2-sgRNA pilot experiment (two-sided Wilcoxon rank-sum test with Benjamini-Hochberg adjustment). See also **Table S1A**.

C. Differentially expressed *T. gondii* genes for sg(MYR1)-expressing cells compared to sg(UPRT)-expressing cells in the 2-sgRNA pilot experiment (two-sided Wilcoxon rank-sum test with Benjamini-Hochberg adjustment). See also **Table S1B**.

D. Number of single cell transcriptomes recovered for each target gene in 24-sgRNA pilot experiment.

E. Distribution of sg(UPRT)-expressing cells in UMAP of host cell gene expression in 24-sgRNA pilot experiment.

F. Distribution of cells expressing sgRNAs targeting select effectors in UMAP of host cell gene expression in 24-sgRNA pilot experiment.

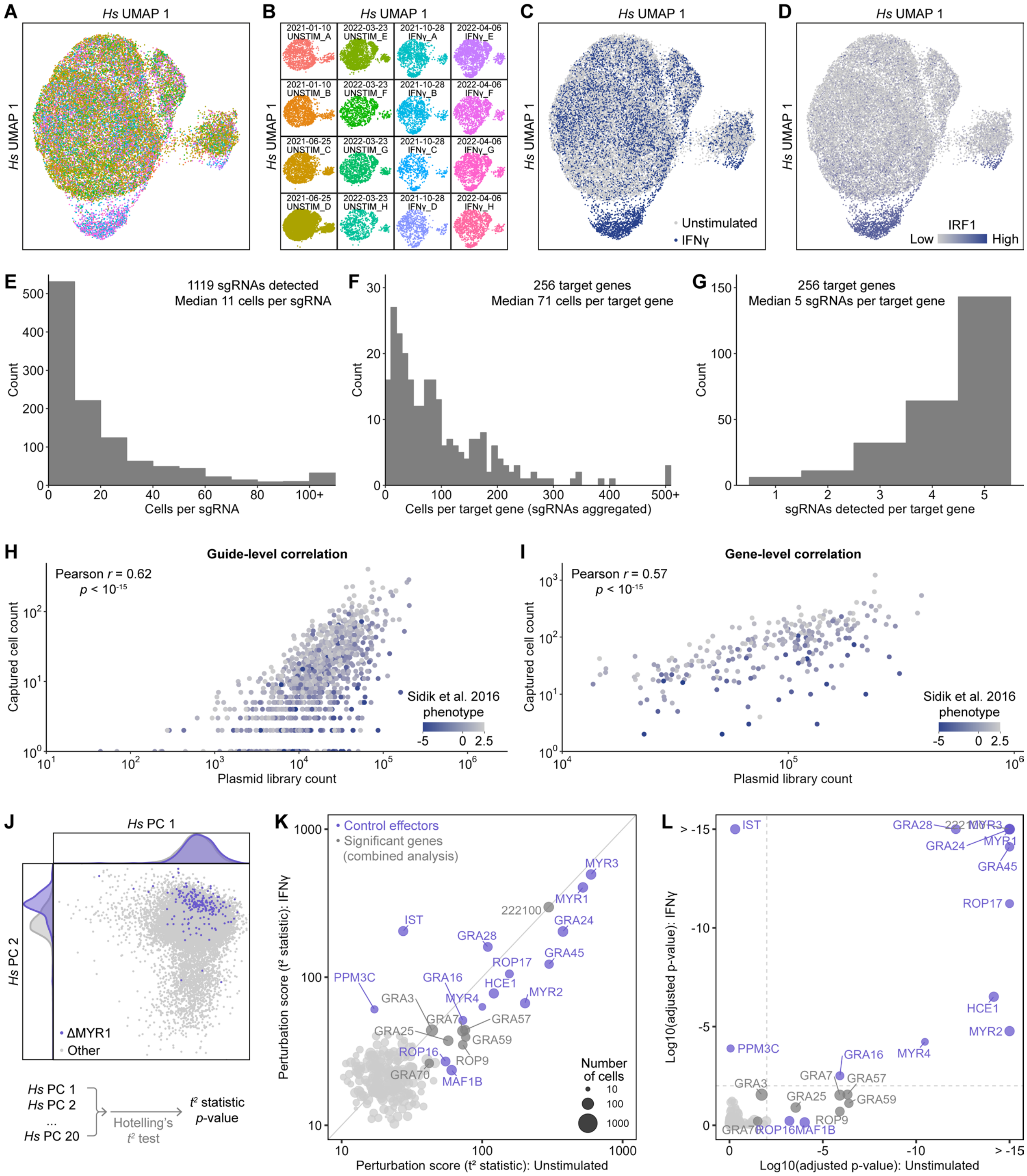

Figure S3. Quality control analysis of dual perturb-seq screen, related to Figure 2.

A. UMAP of single cell transcriptomes based on host cell gene expression coloured by sample.

B. UMAP of single cell transcriptomes split by sample.

C. UMAP of single cell transcriptomes coloured by condition (unstimulated or stimulated with IFNγ).

D. Expression of the interferon-stimulated gene IRF1.

E. Histogram of the number of single cell transcriptomes expressing each sgRNA. See also **Table S3**.

F. Histogram of the number of single cell transcriptomes for each target gene.

G. Histogram of the number of sgRNAs detected for each target gene.

H. Correlation between the number of read counts in bulk sequencing data of perturb-seq plasmid library and the number of single cell transcriptomes for each sgRNA. See also **Table S3**.

I. Correlation between the number of read counts in bulk sequencing data of perturb-seq plasmid library and the number of single cell transcriptomes summed for each target gene.

J. Illustration of Hotelling's t^2-test on single cell PCA embeddings.

K. Correlation between perturbation scores (Hotelling's t^2-test statistic) in unstimulated and IFNγ-stimulated samples. See also **Table S4**.

L. Correlation between Hotelling's t^2-test p-values in unstimulated and IFNγ-stimulated samples. See also **Table S4**.

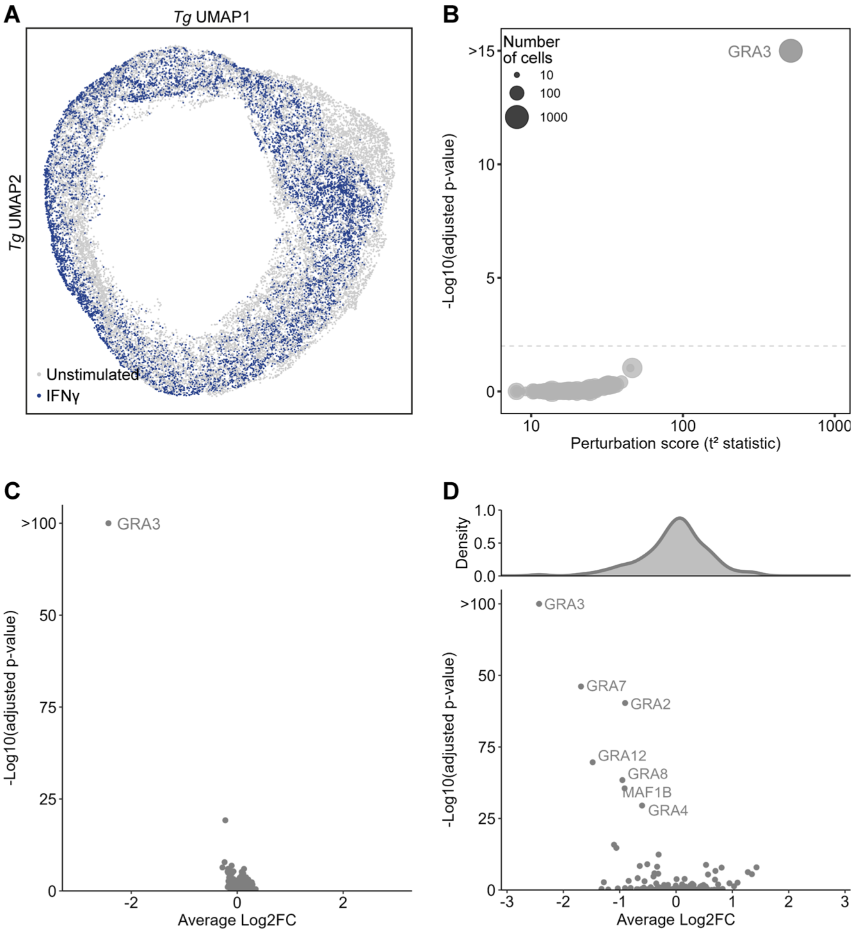

Figure S4. Perturbation of the *T. gondii* transcriptome by effector proteins, related to Figure 2.

A. UMAP of single cell transcriptomes based on *T. gondii* gene expression with cells coloured by condition (unstimulated or stimulated with IFNγ).

B. Perturbation of *T. gondii* transcriptome by effectors, measured by Hotelling's t^2 test on PCA embeddings of single cell transcriptomes with Benjamini Hochberg adjustment. See also **Table S5**.

C. Differentially expressed *T. gondii* genes for sg(GRA3)-expressing cells compared to all other cells (two-sided Wilcoxon rank-sum test with Benjamini-Hochberg adjustment). See also **Table S6**.

D. Differential expression of *T. gondii* genes targeted in this screen in cells expressing the cognate sgRNA that were detectably expressed in at least 25% of non-perturbed cells, with smoothed density estimate of average Log2FCs. See also **Table S7**.

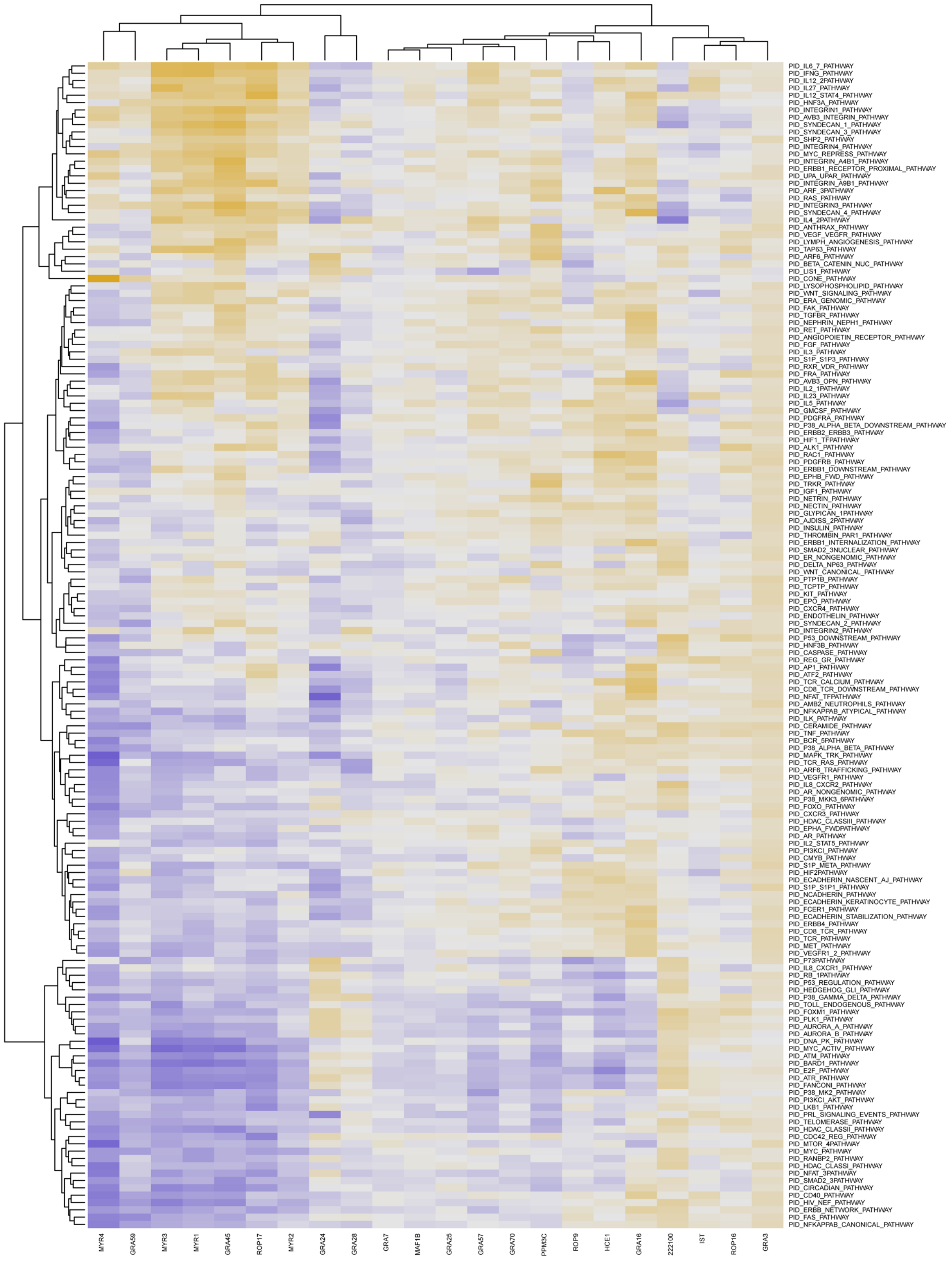

Figure S5. Pathway Interaction Database gene sets that are significantly differentially regulated by *T. gondii* effector proteins, related to Figure 2.

Average VISION signature scores of Pathway Interaction Database gene sets that are significantly differentially regulated by at least one significant effector ($p < 0.01$, two-sided Wilcoxon rank-sum test with Benjamini-Hochberg adjustment). See also **Table S8**.

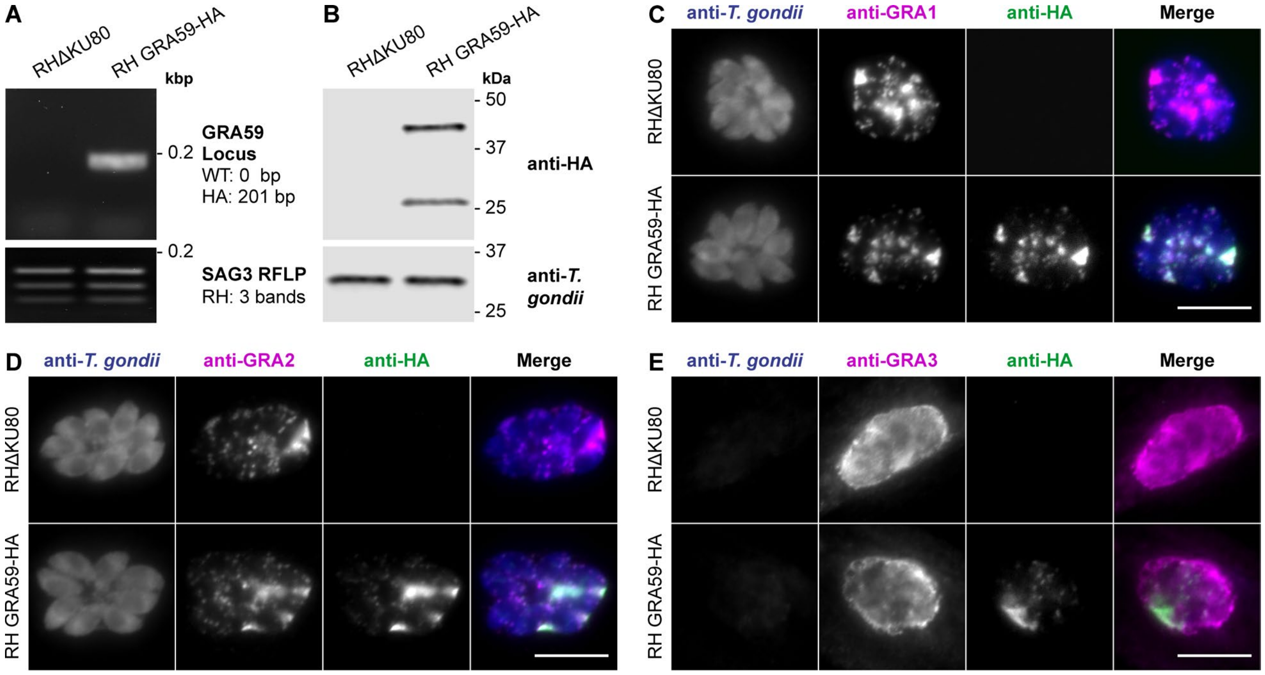

Figure S6. C-terminal epitope tagging of GRA59, related to Figure 3.

A. Verification of HA tagging by diagnostic PCR.

B. Verification of GRA59-HA expression by Western blot.

C. Co-localisation of GRA59-HA with GRA1 by immunofluorescence assay. Scale bar = 10 μm.

D. Co-localisation of GRA59-HA with GRA2 by immunofluorescence assay. Scale bar = 10 μm.

E. Co-localisation of GRA59-HA with GRA3 by immunofluorescence assay in cells permeabilised with 0.1% saponin for 15 min. Scale bar = 10 μm.

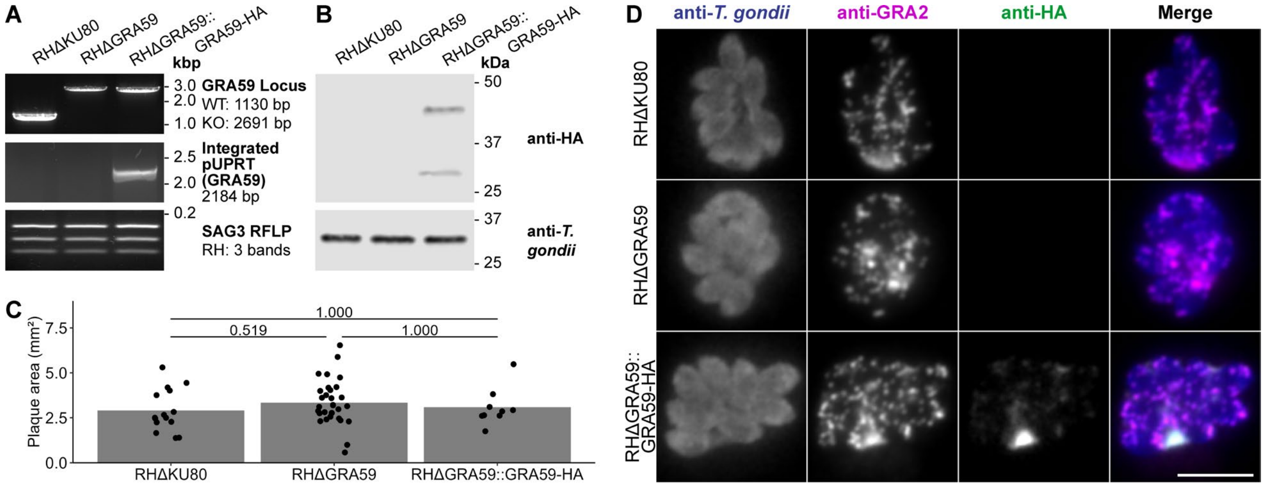

Figure S7. Knockout and complementation of GRA59, related to Figure 3.

A. Verification of GRA59 knockout and complementation by diagnostic PCR.

B. Verification of GRA59-HA expression by Western blot.

C. Plaque assay for RHΔKU80, RHΔGRA59, and RHΔGRA59::GRA59-HA. One biological replicate; points represent individually measured plaques. Differences tested by two-sided *t*-test with Bonferroni correction.

D. Verification of GRA59-HA expression and localisation by immunofluorescence assay. Scale bar = 10 μm.

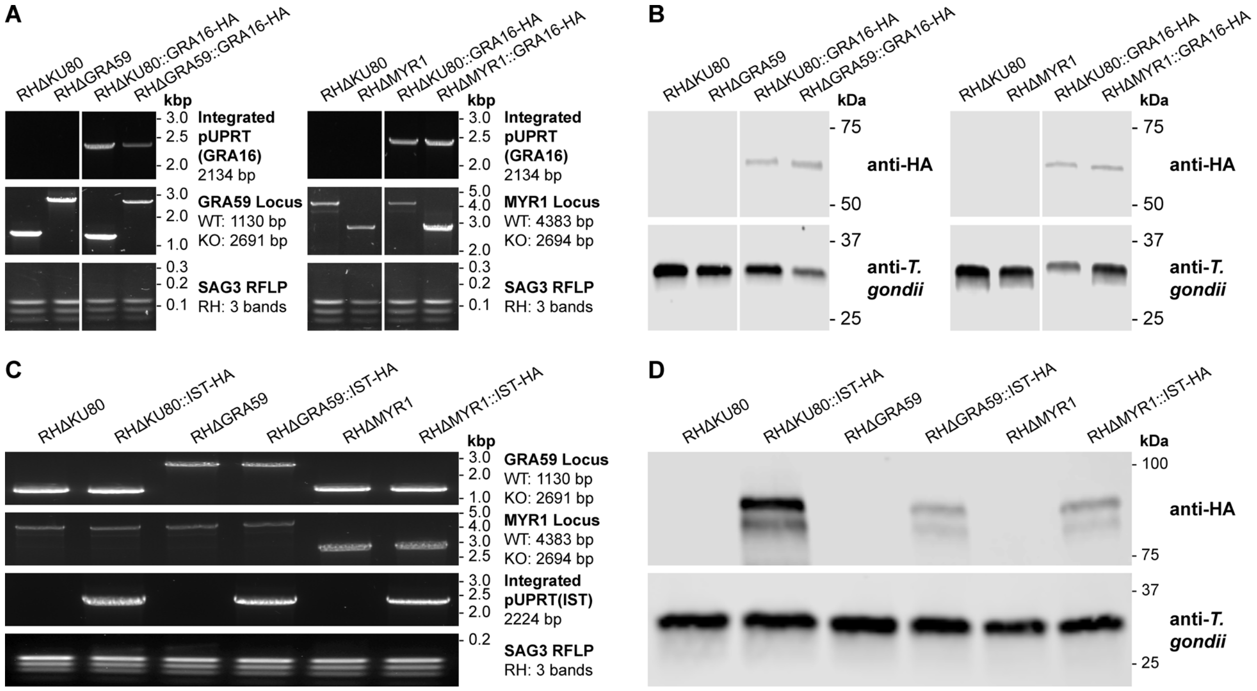

Figure S8. Introduction of GRA16-HA to GRA59 and MYR1 knockout cell lines, related to Figure 3.

A. Verification of pUPRT(GRA16-HA) integration by diagnostic PCR.

B. Verification of GRA16-HA expression by Western blot.

C. Verification of pUPRT(IST-HA) integration by diagnostic PCR.

D. Verification of IST-HA expression by Western blot.

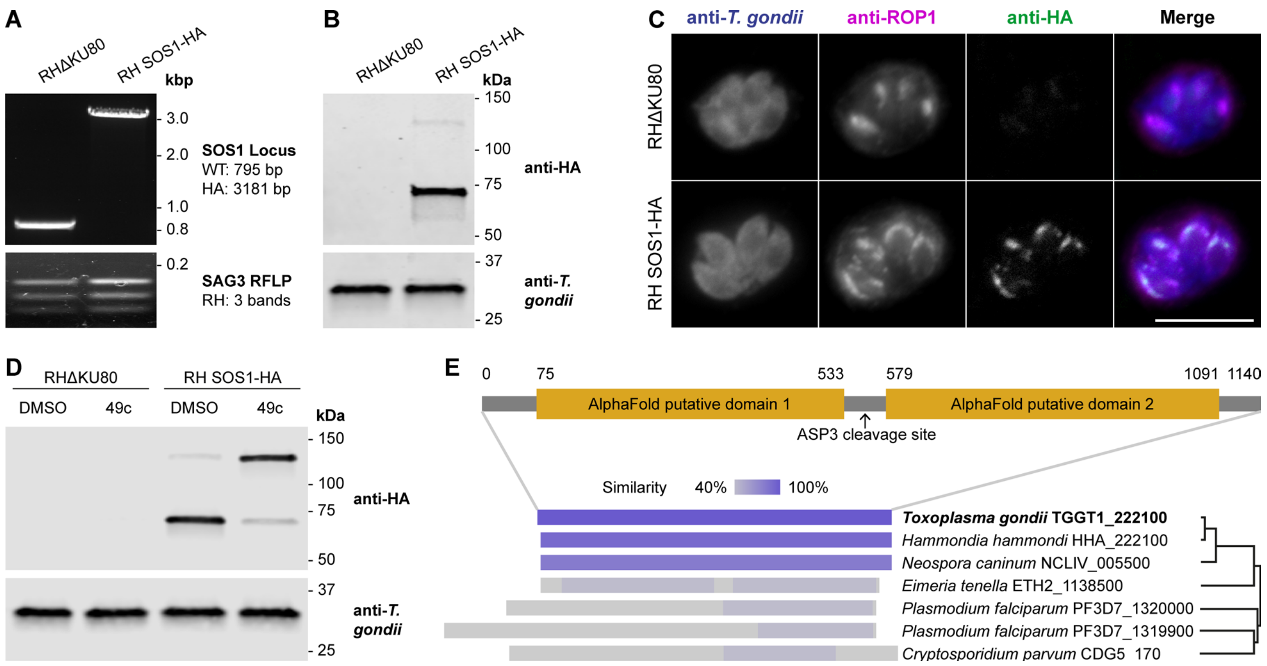

Figure S9. C-terminal epitope tagging of SOS1, related to Figure 4.

A. Verification of HA tagging by diagnostic PCR.

B. Verification of SOS1-HA expression by Western blot.

C. Co-localisation of SOS1-HA with ROP1 by immunofluorescence assay. Scale bar = 10 µm.

D. Treatment of parasites with the ASP3 inhibitor 49c reduces processing of SOS1. 10 µM 49c was added at 1 hpi and parasites were harvested by syringe-lysis at 48 hpi.

E. Putative structure of SOS1 and alignment to homologues detected in Apicomplexa.

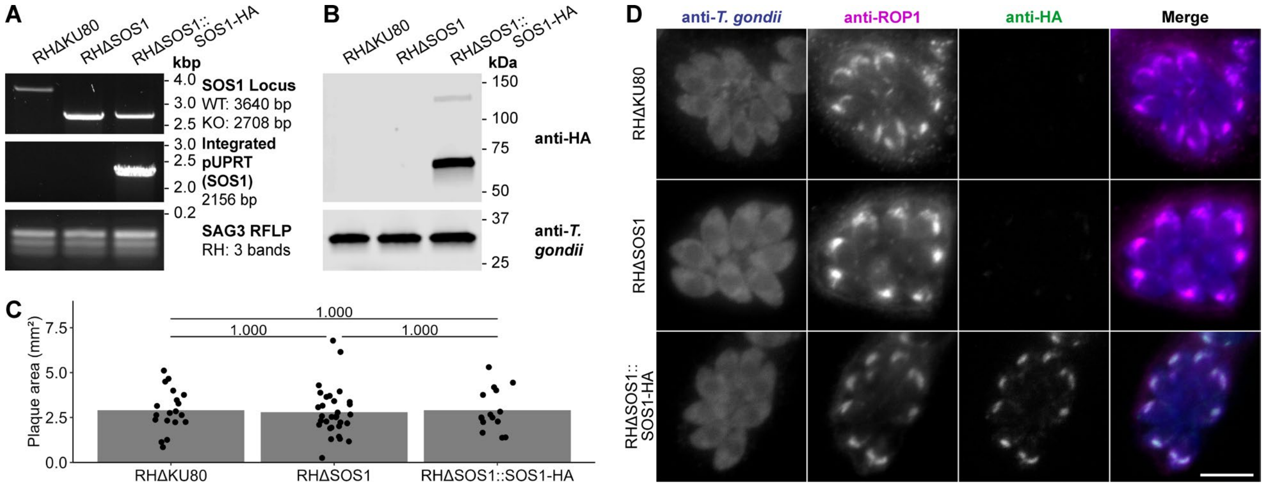

Figure S10. Knockout and complementation of SOS1 in *T. gondii* RH, related to Figure 4.

A. Verification of SOS1 knockout and complementation by diagnostic PCR.

B. Verification of SOS1-HA expression by Western blot.

C. Plaque assay for RHΔKU80, RHΔSOS1, and RHΔSOS1::SOS1-HA. One biological replicate; points represent individually measured plaques. Differences tested by two-sided *t*-test with Bonferroni correction.

D. Verification of SOS1-HA expression by immunofluorescence assay. Scale bar = 10 μm.

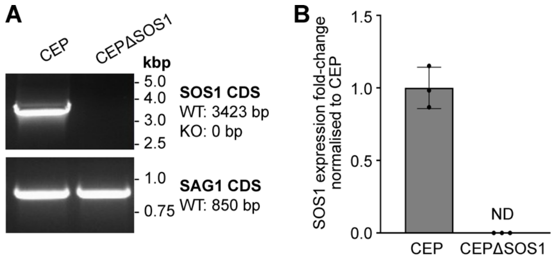

Figure S11. Knockout and complementation of SOS1 in *T. gondii* CEP, related to Figure 5.

A. Verification of SOS1 knockout by diagnostic PCR.

B. qPCR quantification of SOS1 mRNA expression relative to *Tg*Actin.
