# Supplementary material for: High-throughput identification of pathogen effector proteins that target host transcription by dual perturb-seq
Source: Cell Host Microbe. Author manuscript; Available in PMC 2026 Apr 9. (PMC12033024; doi:10.1016/j.chom.2023.09.003)
Supplement: Suppl files [file EMS212870-supplement-Suppl_files.zip › 1-s2.0-S1931312823003700-mmc13.pdf]

Resource

Cell Host & Microbe

High-throughput identification of *Toxoplasma gondii* effector proteins that target host cell transcription

Graphical abstract

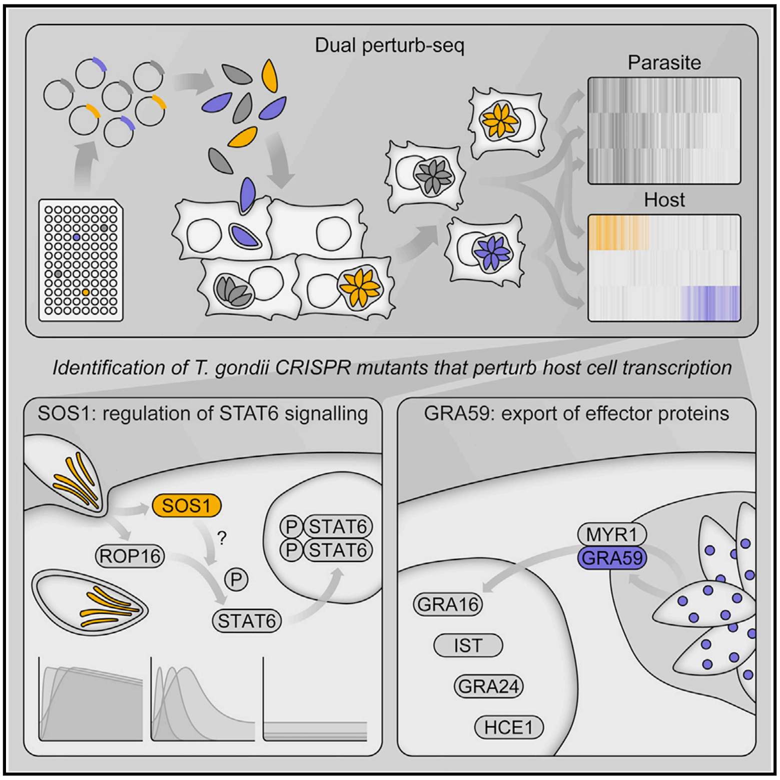

Authors

Simon Butterworth, Kristina Kordova, Sambamurthy Chandrasekaran, ..., Robert Goldstone, Anita A. Koshy, Moritz Treeck

Correspondence

mtreeck@igc.gulbenkian.pt

In brief

Many intracellular pathogens secrete effector proteins that modify host cell transcription for the benefit of the pathogen. By combining pooled CRISPR knockout screening in the pathogen *Toxoplasma gondii* with single-cell RNA sequencing of *T. gondii*-infected cells, Butterworth et al. are able to identify these effectors in an unbiased, high-throughput manner.

Highlights

- Pooled CRISPR screening with a dual host-pathogen single-cell transcriptome readout
- Identification of *Toxoplasma gondii* effector proteins that target host transcription
- *Tg*GRA59 contributes to export of dense granule effectors into the host cell
- *Tg*SOS1 is necessary for sustained host STAT6 signaling

Butterworth et al., 2023, Cell Host & Microbe *31*, 1748–1762
October 11, 2023 © 2023 The Author(s). Published by Elsevier Inc.
https://doi.org/10.1016/j.chom.2023.09.003

CellPress
OPEN ACCESS

Cell Host & Microbe

Resource

High-throughput identification of *Toxoplasma gondii* effector proteins that target host cell transcription

Simon Butterworth,[1] Kristina Kordova,[1] Sambamurthy Chandrasekaran,[2] Kaitlin K. Thomas,[2] Francesca Torelli,[1] Eloise J. Lockyer,[1] Amelia Edwards,[3] Robert Goldstone,[3] Anita A. Koshy,[2,4,5] and Moritz Treeck[1,6,7,*]
[1]Signalling in Apicomplexan Parasites Laboratory, The Francis Crick Institute, London NW1 1AT, UK
[2]BIO5 Institute, University of Arizona, Tucson, AZ 85719, USA
[3]Advanced Sequencing Facility, The Francis Crick Institute, London NW1 1AT, UK
[4]Department of Immunobiology, University of Arizona, Tucson, AZ 85719, USA
[5]Department of Neurology, University of Arizona, Tucson, AZ 85719, USA
[6]Cell Biology of Host-Pathogen Interaction Laboratory, Instituto Gulbenkian de Ciência, Oeiras 2780-156, Portugal
[7]Lead contact
*Correspondence: mtreeck@igc.gulbenkian.pt
https://doi.org/10.1016/j.chom.2023.09.003

SUMMARY

Intracellular pathogens and other endosymbionts reprogram host cell transcription to suppress immune responses and recalibrate biosynthetic pathways. This reprogramming is critical in determining the outcome of infection or colonization. We combine pooled CRISPR knockout screening with dual host-microbe single-cell RNA sequencing, a method we term dual perturb-seq, to identify the molecular mediators of these transcriptional interactions. Applying dual perturb-seq to the intracellular pathogen *Toxoplasma gondii*, we are able to identify previously uncharacterized effector proteins and directly infer their function from the transcriptomic data. We show that *Tg*GRA59 contributes to the export of other effector proteins from the parasite into the host cell and identify an effector, *Tg*SOS1, that is necessary for sustained host STAT6 signaling and thereby contributes to parasite immune evasion and persistence. Together, this work demonstrates a tool that can be broadly adapted to interrogate host-microbe transcriptional interactions and reveal mechanisms of infection and immune evasion.

INTRODUCTION

Endosymbiosis, a phenomenon in which one organism resides within the cells of another, is widespread across all domains of life and spans a continuum from mutually beneficial to pathogenic.[1] Establishment of endosymbiosis commonly involves transcriptional reprogramming of the host cell to downregulate immune responses and maintain a favorable environment for the endosymbiont.[2] Modulation of the host cell is often mediated by effector proteins secreted by the endosymbiont that directly interface with host signaling pathways.[3,4]

The single-celled eukaryotic parasite *Toxoplasma gondii* is outstanding in both the number of identified effector proteins that target host cell transcription and breadth of this reprogramming. Whereas bacterial effectors are exported from the cytosol through molecular secretion systems, *T. gondii* delivers effector proteins into the host cell from two types of secretory organelles, rhoptries and dense granules. Rhoptry proteins are injected directly into the host cytoplasm during parasite invasion of a host cell. Dense granule proteins are secreted into the parasitophorous vacuole in which *T. gondii* resides following host cell invasion. Some secreted dense granule proteins are then transported into the host cell via a secondary export mechanism.[5] More than 200 proteins have been localized to the rhoptries and dense granules of *T. gondii*, of which the vast majority are uncharacterized.[6] Most of these proteins represent novel ortholog groups found only in the Apicomplexa phylum of intracellular parasites or sub-groups thereof. Many do not contain recognizable conserved functional domains that would inform on function.[6] Linking *T. gondii*-induced transcriptional responses in the host cell to specific effector proteins is thus highly challenging. Moreover, existing competitive growth-based pooled knockout screens in *T. gondii* have failed to identify effector proteins known to target host cell transcription, despite their critical roles in parasite virulence, indicating that a different methodology is needed.[7–10]

Here, we adapt a method for pooled CRISPR-Cas9 knockout screening with a single-cell transcriptome readout to measure the impact of gene knockouts in an intracellular pathogen on the infected host cell, which we term dual perturb-seq. We demonstrate this method in *T. gondii* using a plasmid vector that enables the direct capture of both mRNAs and sgRNAs.

 © 2023 The Author(s). Published by Elsevier Inc.
This is an open access article under the CC BY license (http://creativecommons.org/licenses/by/4.0/).

CellPress
OPEN ACCESS

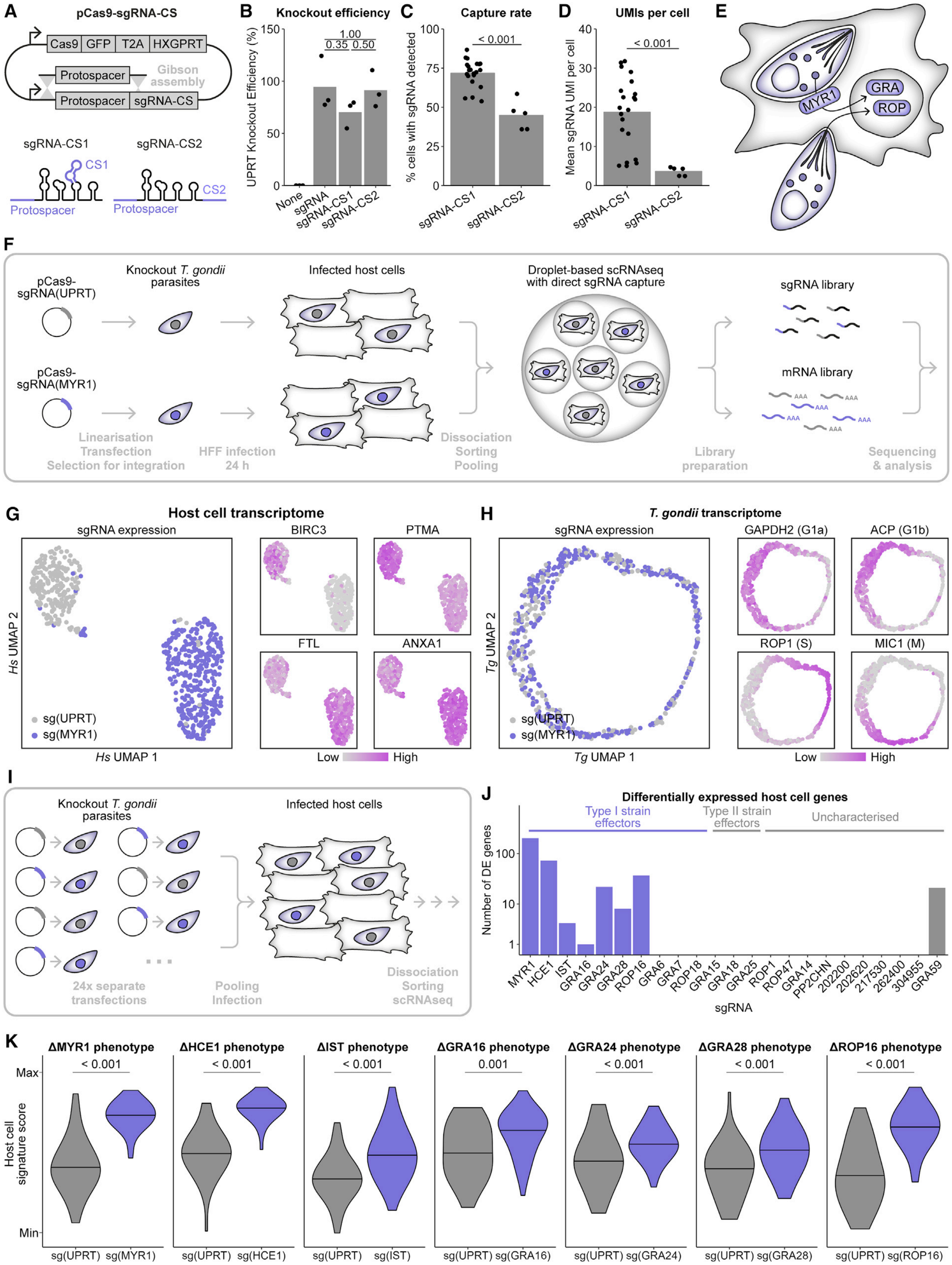

(legend on next page)

Single-cell transcriptomic profiles of host cells infected with genetically perturbed parasites accurately recapitulate bulk RNA sequencing = phenotypes of established effector proteins. We screen a library of >1,000 sgRNAs targeting 256 parasite genes encoding secreted rhoptry and dense granule proteins to gain a system-level view of host cell reprogramming by the *T. gondii* secretome. This screen identifies all previously known effector proteins that modulate host cell transcription and additional putative effectors. We validate the dual perturb-seq phenotypes for two such effectors, showing that GRA59 contributes to export of dense granule effectors into the host cell and that the previously unknown effector SOS1 is essential for sustained STAT6 signaling and M2 polarization of infected cells.

RESULTS

Optimization of a direct-capture perturb-seq vector for *T. gondii*

Single-cell CRISPR screening methods require identification of the sgRNA protospacer sequence to assign a perturbation identity to each cell. In direct-capture perturb-seq, the insertion of a "capture sequence" allows for direct sequencing of the sgRNA transcript in addition to the polyadenylated mRNA transcriptome following droplet-based cell partitioning and molecular barcoding.[11]

To enable direct-capture perturb-seq in *T. gondii*, we modified our existing Cas9-sgRNA plasmid vector[7] by introducing one of the two validated sgRNA-capture sequence configurations, CS1 and CS2[11] (Figure 1A). Following transfection of these vectors into *T. gondii* by electroporation, we did not find any significant difference in knockout efficiency of the non-essential *T. gondii* UPRT gene for either sgRNA compared with the unmodified sgRNA, as measured by knockout-induced resistance to the toxic nucleoside analog 5-fluorodeoxyuridine[12] (Figure 1B). As sgRNA-CS2 had a higher mean knockout efficiency than sgRNA-CS1, we used this configuration for our initial validation experiment.

However, across all experiments we completed as part of this work, we found that the capture rate of sgRNA-CS1 was significantly better than sgRNA-CS2 in terms of the percentage of cells in which any sgRNA is detected (Figure 1C) and the number of sgRNA unique molecular identifiers (UMIs) detected (Figure 1D). Therefore, in later experiments, we used sgRNA-CS1 to maximize the number of recovered cells with an identifiable knockout.

Dual perturb-seq transcriptional profiles recapitulate phenotypes of known *T. gondii* effectors

As perturb-seq has not been previously attempted in a non-mammalian system, we carried out two experiments to validate the dual perturb-seq transcriptional profiles of host cells infected with *T. gondii* mutants. First, we targeted the *T. gondii* UPRT gene, as a negative control, and the MYR1 gene, as a positive control. MYR1 is essential for export of dense granule effectors into the host cell, and it has been shown that knockout of MYR1 abrogates the majority of host cell transcriptional changes induced by *T. gondii*[15,16] (Figure 1E). *T. gondii* RHΔHXGPRT parasites were transfected with perturb-seq plasmid vectors targeting either UPRT or MYR1, then used to infect human foreskin fibroblasts (HFFs) for 24 h at a multiplicity of infection (MOI) of 0.1 (Figure 1F). Infected cells were sorted based on Cas9-GFP expression in the intracellular parasites and pooled for single-cell RNA-seq (scRNA-seq) using the 10x Genomics Chromium platform.

Figure 1. Dual perturb-seq transcriptional profiles recapitulate phenotypes of known *T. gondii* effectors

(A) Schematic of perturb-seq plasmid vector and capture sequence sgRNAs.

(B) Knockout efficiency of perturb-seq vectors. *T. gondii* parasites were transfected with perturb-seq vectors targeting the UPRT gene and selected for integration of the plasmid using the HXGPRT marker. Loss of function of the UPRT gene was measured by plaque assay in the presence/absence of 5-fluorodeoxyuridine, with the knockout efficiency calculated as the percentage of plaque-forming units resistant to 5-fluorodeoxyuridine. Differences were tested by two-sided t test with Bonferroni correction.

(C) Percentage of infected cells with a detectable sgRNA in all dual perturb-seq samples. Difference tested by two-sided t test.

(D) Mean number of sgRNA UMIs in cells with a detectable sgRNA in all dual perturb-seq samples. Difference tested by two-sided t test.

(E) Schematic of *T. gondii* effector protein export into the host cell. Effector proteins may be secreted into the host cell from the rhoptry organelles during parasite invasion of the host cell or may be secreted into the parasitophorous vacuole of intracellular parasites and subsequently translocated into the host cell by a complex that contains the MYR1 protein.

(F) Experimental strategy for dual perturb-seq experiment with two sgRNAs. *T. gondii* parasites were transfected with perturb-seq vectors targeting either UPRT or MYR1 and selected for integration of the vector into the genome. The transfected parasites were used to infect human foreskin fibroblasts (HFFs) for 24 h, following which the infected cells were purified by FACS and analyzed by droplet-based scRNA-seq with capture of both polyadenylated host and parasite mRNAs and the sgRNA transcripts.

(G) UMAP of single-cell transcriptomes based on host cell gene expression and colored according to the sgRNA species detected or by expression of the most significantly upregulated and downregulated host cell genes in sg(MYR1)-expressing cells.

(H) UMAP of single-cell transcriptomes based on *T. gondii* gene expression and colored according to the sgRNA species detected or by expression of *T. gondii* cell cycle marker genes.[13] Expression of glycolytic enzymes (e.g., GAPDH2) peaks in early G1 phase (G1a), whereas apicoplast-localized proteins (e.g., ACP) peak in late G1 phase (G1b), rhoptry proteins (e.g., ROP1) in S phase, and microneme proteins (e.g., MIC1) in M phase. Note that *T. gondii* lacks a discernible G2 phase.[14]

(I) Experimental strategy for dual perturb-seq experiment with 24 sgRNAs. *T. gondii* parasites were transfected with one of the 24 perturb-seq vectors and selected for integration of the vector into the genome. The knockout parasites were pooled together to infect HFFs for 24 h and infected cells purified and analyzed by scRNA-seq as above.

(J) For each sgRNA in the 24-sgRNA experiment, the number of significantly differentially expressed host cell genes was determined relative to sg(UPRT) (adjusted p value < 0.01 and average log_2-fold change > 0.5).
See also Table S2.

(K) Single-cell transcriptomes in the 24-sgRNA experiment were scored for expression of marker genes regulated by established effector proteins using VISION. A higher score indicates greater concordance with published bulk RNA-seq data for a given effector protein. Differences were tested by two-sided Wilcoxon rank-sum test.

CellPress
OPEN ACCESS

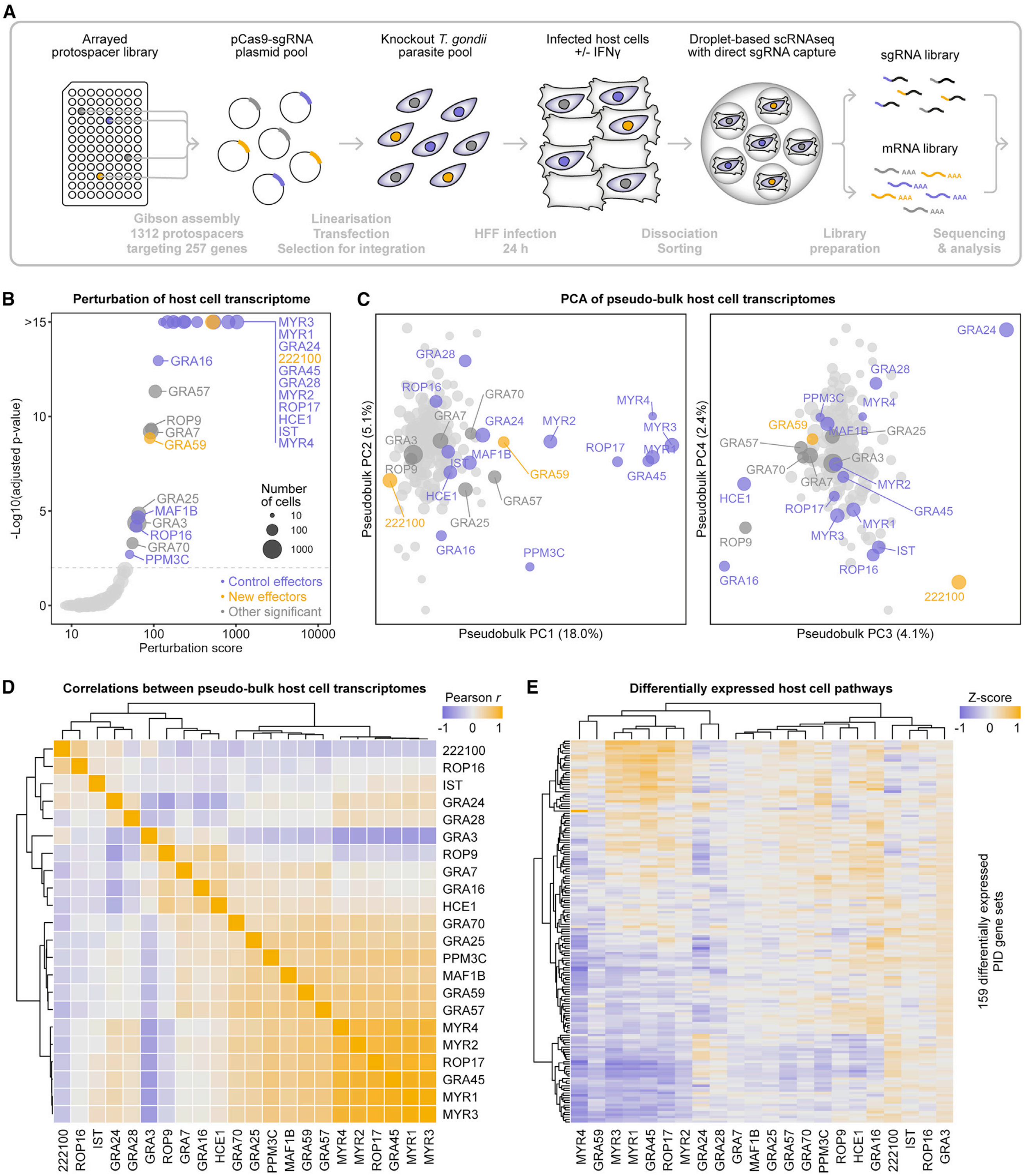

Figure 2. A dual-perturb-seq screen identifies *T. gondii* effectors that target host cell transcription

(A) Schematic of dual perturb-seq screen. Single-stranded DNA (ssDNA) oligonucleotides encoding protospacer sequences were selected from an arrayed library and incorporated into the perturb-seq vector by pooled Gibson assembly. *T. gondii* parasites were transfected with the perturb-seq vector pool and selected for integration of the plasmid into the genome. HFFs were infected with the resulting pool of knockout parasites for 24 h with or without IFNγ stimulation. Infected cells were purified by FACS and analyzed by scRNA-seq.

(legend continued on next page)

Sequencing reads were aligned to a dual *H. sapiens* and *T. gondii* reference, and the cell barcodes were filtered to retain only infected cells in which a single sgRNA species was detected (Figures S1 and S2A). Inspecting these single-cell transcriptomes by uniform manifold approximation projection (UMAP) using only the host cell gene expression, we observed that cells expressing the MYR1-targeting sgRNA formed a distinct cluster from those expressing the UPRT-targeting sgRNA, indicating that knockout of these genes in the intracellular parasite results in differing transcriptional profiles in the host cell (Figure 1G). 1,234 host cell genes were significantly differentially expressed with a $\log_2$-fold change greater than 0.5 between cells expressing the MYR1-targeting and UPRT-targeting sgRNAs, including many previously shown to be dependent on MYR1[16] (Figures 1G and S2B; Table S1A).

In each infected host cell, we were also able to resolve the transcriptome of the synchronous intracellular parasites. Projecting the cells by UMAP using the *T. gondii* gene expression, we found that the transcriptomes resolved in a circular structure that recapitulated the parasite cell cycle (Figure 1H). Very few *T. gondii* genes were differentially expressed between cells expressing the MYR1-targeting and UPRT-targeting sgRNAs, indicating that knockout of these genes does not induce broad changes in the parasite transcriptome (Figure S2C; Table S1B).

To further validate this dual perturb-seq approach for identifying *T. gondii* effector proteins, we generated a panel of 22 vectors targeting 12 effector proteins that have been shown to impact host cell transcription and 10 rhoptry and dense granule proteins for which the host cell transcriptome has not been characterized. We transfected *T. gondii* RHΔHXGPRT separately with the 22 new vectors and the MYR1- and UPRT-targeting vectors and pooled the resulting transfectants into a single mixed population. This parasite pool was used to infect HFFs for 24 h, following which infected cells were enriched by fluorescence-activated cell sorting (FACS) for scRNA-seq as before (Figure 1I). We recovered between 10 and 72 single-cell transcriptomes for each sgRNA (Figure S1D). Cells expressing different sgRNAs did not segregate into distinct clusters in a UMAP of host gene expression, although different sgRNAs were enriched in different regions of a large main cluster (Figures S2E and S2F).

We identified differentially expressed host genes for seven out of ten effector proteins that have been validated in the highly virulent type I *T. gondii* RH strain used in this experiment: MYR1,[16] HCE1/TEEGR,[17,18] IST,[19,20] GRA16,[21] GRA24,[22] GRA28,[23,24] and ROP16[25] (Figure 1J; Table S2). The numbers of differentially expressed genes for each effector were relatively low, indicating a loss of statistical power when fewer than 100 transcriptomes are recovered per target and that pathway-level rather than gene-level analyses may be more suitable. We did not find any significantly differentially expressed genes for the effectors GRA6,[26] GRA7,[27] or ROP18.[28] These were among the genes for which we recovered the fewest single-cell transcriptomes and so have less statistical power; however, there is also less evidence that these effectors induce changes in the host cell transcriptome during a physiological infection. There were no differentially expressed host genes for the type II *T. gondii* strain-specific effector GRA15,[29] as expected, nor for GRA18[30] or GRA25,[31] suggesting that these are also type II-specific effectors. Of the uncharacterized genes included in this experiment, we identified differentially expressed host genes only for GRA59, which we investigate further later in this study.

Bulk transcriptomic datasets of *T. gondii* knockout strain-infected versus wild-type-infected host cells are available for the seven type I effectors for which we can identify differentially expressed genes, but not for GRA6, GRA7, or ROP18 (see STAR Methods). To determine whether the host cell phenotypes derived from this dual perturb-seq experiment concur with these datasets, we scored the single-cell transcriptomes for the expression of marker genes identified in these prior studies using the VISION package.[32] Cells expressing the sgRNA targeting each of these seven effectors (MYR1, HCE1, IST, GRA16, GRA24, GRA28, and ROP16) had significantly higher scores for the cognate bulk transcriptome signatures compared with sg(UPRT)-expressing cells (Figure 1K). Thus, we show that dual perturb-seq is able to deconvolve the phenotypes of host cells infected with a pool of different *T. gondii* knockouts and that the resulting transcriptomic profiles concur with all available prior datasets.

Identification of *T. gondii* genes that alter host cell transcription through a dual perturb-seq screen

Having validated the dual perturb-seq workflow, we set up a knockout screen to identify all *T. gondii* effectors that target host cell transcription. We selected 256 rhoptry and dense granule-localized proteins comprising the *T. gondii* effectome, based on spatial proteomics data[6] and community annotation.[33] Five protospacer sequences targeting each of these genes were selected from our arrayed library[7] and incorporated into the *T. gondii* perturb-seq vector by pooled Gibson assembly (Table S3). The plasmid pool was transfected into *T. gondii* RHΔHXGPRT, and the resulting pool of knockout parasites was used to infect HFFs (Figure 2A). We carried out the dual perturb-seq screen in both unstimulated and interferon-gamma (IFNγ)-stimulated HFFs to identify effector proteins that modulate the expression of IFNγ-stimulated genes.[19,20,34]

We recovered a total of 25,185 single-cell transcriptomes of HFFs infected with the pool of *T. gondii* mutants (15,920 unstimulated and 9,265 IFNγ-stimulated) (Figures S3A and

(B) Perturbation of host cell transcriptomes by *T. gondii* effectors. Single-cell transcriptomes were analyzed by principal-component analysis (PCA) using the host cell gene expression, and each *T. gondii* effector was tested for an altered distribution of PCA embeddings using Hotelling's t^2 test with Benjamini-Hochberg adjustment.
See also Table S4.
(C) PCA embeddings of pseudo-bulk host cell transcriptomes.
(D) Pearson correlations between pseudo-bulk host cell transcriptomes of significant effectors.
(E) Host cell pathways regulated by putative effectors. Single-cell transcriptomes were scored for expression of gene sets from the PID using VISION. Gene sets that were significantly differentially regulated by at least one putative effector ($p < 0.01$, two-sided Wilcoxon rank-sum test with Benjamini-Hochberg adjustment) are plotted with the mean VISION signature score transformed to a *Z* score.
See also Figure S5 and Table S8.

CellPress
OPEN ACCESS

S3B). Across all the single-cell transcriptomes, we identified 1,119 sgRNAs with a median of 11 transcriptomes per sgRNA (Figure S3E). All of the 256 *T. gondii* target genes were represented by at least one sgRNA; aggregating all sgRNAs targeting a given gene resulted in a median coverage of 71 transcriptomes per gene (Figure S3F). Most target genes were represented by five different sgRNAs, and 93% of target genes were represented by at least three different sgRNAs (Figure S3G). Both at the individual sgRNA level and when aggregated by the target gene, the number of single-cell transcriptomes recovered was directly proportional to sgRNA representation in the perturb-seq plasmid pool as assessed by bulk sequencing (Table S3), although sgRNAs targeting genes with strongly negative fitness phenotypes[35] tended to be less well represented (Figures S3H and S3I).

To identify *T. gondii* gene knockouts that perturb the host cell transcriptome using a single metric, we analyzed the single-cell transcriptomes by principal-component analysis (PCA) and tested for each target gene whether the distribution of single-cell transcriptomes in PCA space differed from the background distribution using Hotelling's t^2 test, a multivariate generalization of Student's t test[36] (Figure S3J). Combining the data from both unstimulated and IFNγ-stimulated cells, we identified 22 *T. gondii* genes that significantly alter the host cell transcriptome, including all 14 controls (Figure 2B; Table S4). We also analyzed the data from unstimulated and IFNγ-stimulated cells separately but did not identify any significant genes specific to the IFNγ-stimulated condition apart from the controls IST[19,20] and PPM3C[37] (Figures S3K and S3L; Table S4). We identified significant perturbations for both GRA7 and GRA25, despite not identifying any DEGs for these knockouts in our earlier pilot experiment. Potentially, the higher coverage in this main screen in terms of the number of single-cell transcriptomes, and the use of multiple sgRNAs for each target, allows us to identify more subtle phenotypes. Most interestingly, we identified significant perturbations for the following six genes that have not previously been implicated in reprogramming host transcription: TGGT1_222100, GRA57, ROP9, GRA59, GRA3, and GRA70.

We then applied Hotelling's t^2 test to single-cell PCA embeddings using the *T. gondii* transcriptome to test whether deletion of any parasite effector alters parasite gene transcription. We only found a significant perturbation for GRA3 (Figure S4B; Table S5). Inspection of genes differentially regulated in sg(GRA3)-expressing cells revealed that this perturbation was primarily driven by downregulation of GRA3 itself, with no other $\log_2$-fold changes greater than 0.11 (Figure S4C; Table S6). With a few exceptions, we did not observe substantial downregulation of most other *T. gondii* genes targeted in this screen in cells expressing the cognate sgRNA (Figure S4D; Table S7), although transcriptional downregulation is not necessarily expected here as protein loss of function results from mutation or truncation of the amino acid sequence.[38] Thus, these data show that knockout of effector proteins does not appear to have any wider effect on the *T. gondii* transcriptome.

Dual perturb-seq data reveal putative effector protein functions

To further investigate these putative effector proteins, we generated pseudo-bulk transcriptomes by averaging the Z-transformed normalized UMI counts for all target genes represented by at least 30 cells, corresponding to the 25th percentile of target genes. Using PCA, we found that the majority of variance between these pseudo-bulk transcriptomes was driven by known parasite effector proteins (Figure 2C). For example, the first principal component identifies a cluster of all known secreted *T. gondii* genes required for export of dense granule effectors (MYR1–4,[15,16,39,40] ROP17,[41] GRA45,[9,40] and PPM3C[37]; GRA44 is also required but has a very strong negative growth phenotype, therefore we did not recover enough cells to analyze[40,42]), whereas the third principal component separates the dense granule effectors GRA16 and HCE1 from GRA24, indicating that these effectors may drive opposing transcriptional responses in the host cell.

To formally identify these apparent clusters of functionally related effector proteins, we calculated Pearson correlation coefficients between the pseudo-bulk transcriptomes of the effectors that significantly perturb host cell gene expression (Figure 2D). Hierarchical clustering based on these correlation coefficients identified four broad clusters of effector proteins. A cluster of tightly correlated pseudo-bulk transcriptomes contained six proteins that have been shown to be essential for dense granule effector export and which likely constitute the ''core'' export machinery: MYR1–4, ROP17, and GRA45.[9,15,39–41] An additional cluster of dense granule proteins (comprising GRA59, GRA57, MAF1B, GRA25, PPM3C, and GRA70) was moderately correlated with the cluster of essential export proteins, putatively indicating some similarity or overlap in phenotype. A third cluster of five genes contained ROP9, GRA7, and GRA3, together with the effectors HCE1 and GRA16 that have been shown to affect the host cell cycle and metabolism.[17,18,21] Finally, we identified a cluster of five genes we interpret as ''immune regulators'': GRA28, which regulates host cell motility and cytokine expression through interaction with chromatin remodelers,[23,24] GRA24, which activates p38α mitogen-activated protein kinase (MAPK),[22] IST, which inhibits the induction of ISGs by recruiting repressive nucleosome remodelers to STAT1 target genes,[19,20] ROP16, which directly phosphorylates and activates the transcription factors STAT3 and STAT6,[25,43,44] and an uncharacterized gene, TGGT1_222100.

To determine which host cell pathways are differentially regulated by these effector proteins, we scored the single-cell transcriptomes for expression of 196 gene sets from the Pathway Interaction Database (PID)[45,46] using VISION[32] and tested for upregulation or downregulation of these pathways for each putative effector protein by Wilcoxon rank-sum test on the signature scores (Figures 2E and S5; Table S8). This analysis reaffirmed many of the known phenotypes of characterized effector proteins, for example, upregulation of the c-Myc pathway by GRA16,[47] activation of p38α MAPK by GRA24,[22] and suppression of interferon responses by IST.[19,20] Furthermore, inspection of differentially regulated pathways suggested functions for the putative effector proteins newly identified here, such as activation of the E2F pathway by ROP9, indicating a role in regulation of the host cell cycle and induction of an interleukin-4 (IL-4)-like response by TGGT1_222100. Hierarchical clustering of the effectors using mean signature scores for 159 pathways

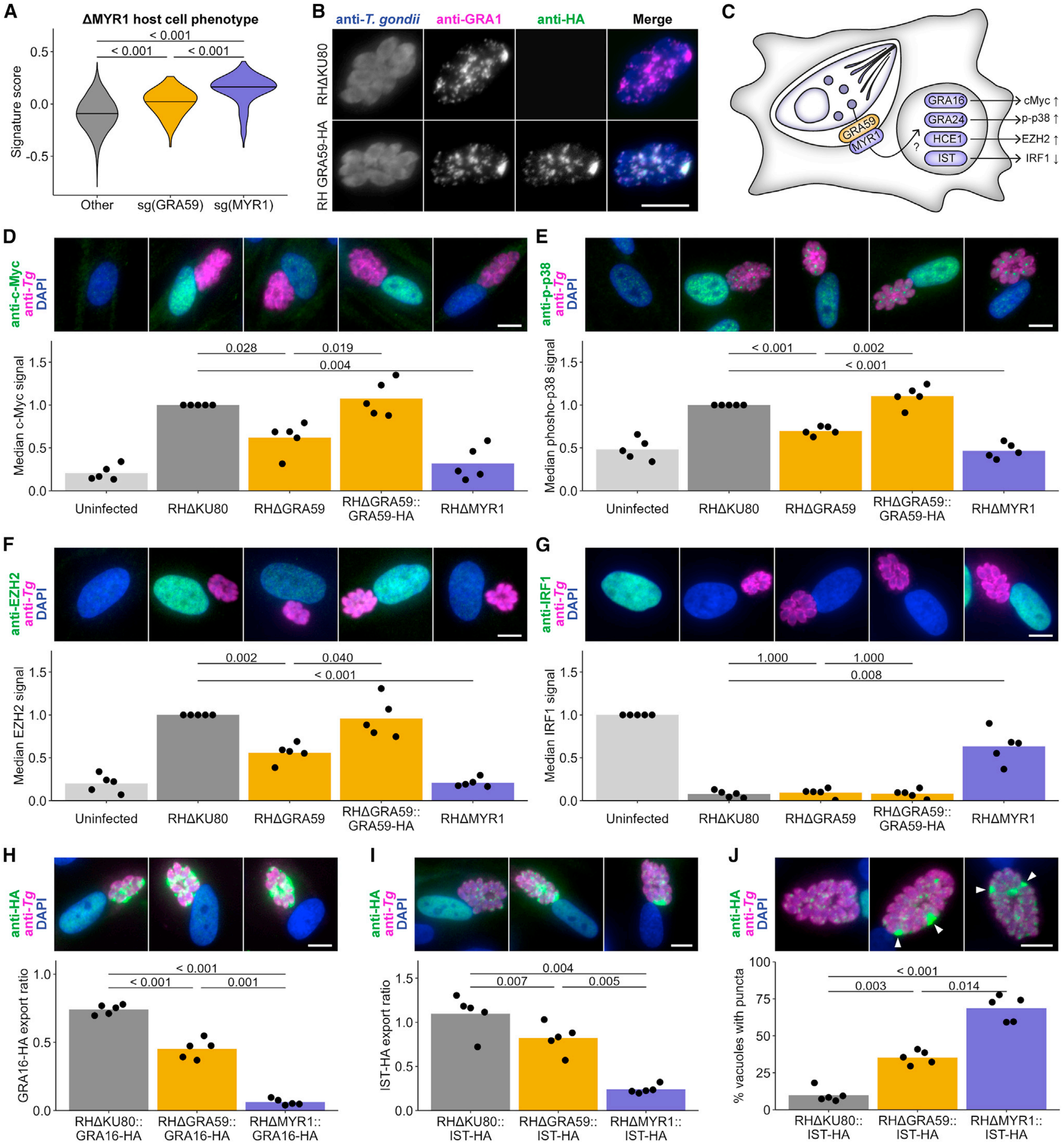

Figure 3. GRA59 (TGGT1_313440) contributes to dense granule effector protein export

(A) Scoring of single-cell transcriptomes for a ΔMYR1-infected phenotype using VISION. Differences tested by two-sided Wilcoxon rank-sum test with Bonferroni adjustment.

(B) Immunofluorescence localization of GRA59-HA within the *T. gondii* vacuole at 24 h post-infection (hpi). Scale bars, 10 μm.

(C) Model of dense granule effector export into the host cell.

(D) Quantification of nuclear c-Myc immunofluorescence in the nuclei of infected cells at 24 hpi. Scale bars, 10 μm. Differences tested by two-sided Wilcoxon rank-sum test with Bonferroni adjustment.

(E) Quantification of nuclear phospho-p38 immunofluorescence in the nuclei of infected cells at 24 hpi. Scale bars, 10 μm. Differences tested by two-sided Wilcoxon rank-sum test with Bonferroni adjustment.

(legend continued on next page)

significantly differentially regulated by at least one effector gave similar results to the clustering using Pearson correlation coefficients.

In summary, with dual perturb-seq, we are able to screen >200 uncharacterized genes in intracellular *T. gondii* parasites for an effect on the transcriptome of mammalian host cells. With a single statistical test, we identify all currently known effector proteins that target host transcription and identify six additional candidate effectors. By inspection of pseudo-bulk transcriptomes by PCA and inter-effector correlation, we identify clusters of functionally related effector proteins. Finally, we are able to determine which host cell pathways are differentially regulated by each effector protein, as a first step toward establishing their function.

GRA59 contributes to export of dense granule effectors

One of the top hits in the dual perturb-seq screen was the dense granule protein GRA59 (TGGT1_313440) (Figure 2B), for which no function is known.[48] Correlation of pseudo-bulk transcriptomes and clustering based on differentially expressed host cell pathways indicated that GRA59 has a similar host cell phenotype to genes that are known to be required for export of dense granule effectors into the host cell (Figures 2D and 2E). By scoring sg(GRA59)-expressing cells for a ΔMYR1-like signature, we found that these cells had an intermediate phenotype compared with sg(MYR1)-expressing cells (Figure 3A).

We tagged GRA59 with a single HA epitope at the C terminus (Figures S6A and S6B) and confirmed prior immunofluorescence localization to the parasitophorous vacuole[48] (Figures S6C–S6E). In fully permeabilized cells, GRA59 co-localized with the vacuole lumen protein GRA1 and the intravacuolar network (IVN)-resident GRA2 (Figures 3B, S6C, and S6D). However, upon partial permeabilization with saponin, which disrupts the host cell plasma membrane but leaves the parasitophorous vacuole membrane (PVM) mostly intact, anti-HA staining was detectable that partially co-localized with the PVM marker GRA3, indicating that the C terminus of GRA59 may be exposed to the host cell cytosol (Figure S6E). In support of this finding, GRA59 was recently shown to be accessible to a host cell-localized biotin ligase.[49] We therefore hypothesized that GRA59 may be a component of the export complex in the PVM (Figure 3C).

We generated GRA59 knockout and complemented cell lines in the RHΔKU80 background (Figure S7) and measured dense granule effector export by quantifying nuclear c-Myc immunofluorescence of infected HFFs. Upregulation of host c-Myc by *T. gondii* is entirely dependent on MYR1-dependent export[15] and partially dependent on the exported effector GRA16.[47] Although the parental RHΔKU80 strain strongly induced host c-Myc and knockout of MYR1 completely ablated this upregulation, as expected, knockout of GRA59 resulted in an intermediate phenotype that was restored by complementation (Figure 3D). This phenotype is consistent either with a role for GRA59 in effector export or in directly upregulating c-Myc in addition to or in partnership with GRA16. We therefore measured three additional host cell phenotypes of MYR-dependent exported effector proteins: phosphorylation and nuclear translocation of p38 MAPK by GRA24,[22] upregulation of EZH2 by HCE1/TEEGR,[17,18] and inhibition of IFNγ-induced IRF1 expression by IST.[19,20] For both p38 phosphorylation and EZH2 upregulation, knockout of GRA59 resulted in a phenotype intermediate between the parental RHΔKU80 strain and RHΔMYR1 similarly to c-Myc upregulation (Figures 3D and 3E). These results indicate that GRA59 contributes to the export of multiple effector proteins but that knockout does not completely abolish export, as is the case for MYR1. In contrast, suppression of IFNγ-mediated IRF1 induction was not affected by GRA59, suggesting normal export of the effector protein IST (Figure 3G).

To directly test whether effector export was affected by GRA59, we introduced an HA-tagged variant of either GRA16 or IST to the RHΔKU80, RHΔGRA59, and RHΔMYR1 strains (Figure S8). Following export to the host cell, both GRA16 and IST localize to the host cell nucleus. Therefore, we quantified the export of GRA16-HA and IST-HA as the ratio of anti-HA immunofluorescence signal in the host cell nuclei compared with that remaining in the vacuole at 24 h post-infection (hpi). In accordance with the c-Myc phenotype, we observed an intermediate reduction in GRA16-HA export by the RHΔGRA59 strain compared with RHΔKU80 and RHΔMYR1 (Figure 3H). Surprisingly, export of IST-HA was also reduced in the RHΔGRA59 strain, although suppression of IRF1 was not affected (Figure 3I). Furthermore, we noticed the presence of accumulations of IST-HA signal within the vacuole of the RHΔGRA59 and RHΔMYR1 strains that were largely absent in RHΔKU80 (Figure 3J). The percentage of vacuoles containing these IST-HA accumulations mirrored the reduced export to the host cell nucleus, indicating retention of IST-HA within the vacuole when export is limited. Nearly all vacuoles, including those of RHΔKU80, contained accumulations of GRA16-HA; therefore, it was not possible to quantify retention of GRA16-HA in the same manner.

Together, these data confirm the apparent phenotype of GRA59 in the dual perturb-seq screen: GRA59 has a MYR1-like phenotype and contributes to the export of dense granule proteins from the vacuole into the host cell. However, unlike for MYR1, knockout of GRA59 does not completely ablate export. Instead, there is an intermediate reduction in export of GRA16 and IST, and indications that export of GRA24 and HCE1 are similarly affected.

(F) Quantification of nuclear EZH2 immunofluorescence in the nuclei of infected cells at 24 hpi. Scale bars, 10 μm. Differences tested by two-sided Wilcoxon rank-sum test with Bonferroni adjustment.

(G) Quantification of nuclear IRF1 immunofluorescence in the nuclei of IFNγ-stimulated infected cells at 24 hpi. Scale bars, 10 μm. Differences tested by two-sided Wilcoxon rank-sum test with Bonferroni adjustment.

(H) Ratio of GRA16-HA immunofluorescence in the host cell nuclei compared with the vacuole at 24 hpi. Scale bars, 10 μm. Differences tested by paired two-sided t test with Bonferroni adjustment.

(I) Ratio of IST-HA immunofluorescence in the host cell nuclei compared with the vacuole at 24 hpi. Scale bars, 10 μm. Differences tested by paired two-sided t test with Bonferroni adjustment.

(J) Percentage of vacuoles containing accumulations of IST-HA immunofluorescence at 24 hpi. White arrowheads indicate accumulations. Scale bars, 10 μm. Differences tested by paired two-sided t test with Bonferroni adjustment.

Along with GRA59, this cluster of putative effectors (Figure 2D) contained the dense granule proteins GRA57 and GRA70, which we and others have recently shown form part of a complex implicated in parasite resistance to innate immune restriction in HFFs.[50,51] The identification of GRA57 and GRA70 in this screen and their apparent similarity to GRA59 was surprising as we have shown that, in contrast to GRA59, GRA57 does not affect induction of host cell c-Myc, a proxy for GRA16 export into the host cell (although this was not investigated for GRA70).[50] Although it is possible that both GRA57 and GRA70 are false-positive hits in this screen due to sgRNA off-target effects, the identification of two members of a complex with apparently similar phenotypes indicates that further investigation of a possible role in host cell reprogramming may be warranted.

SOS1 is required for sustained STAT6 signaling

Aside from established effectors, the gene with the highest perturbation score in the screen was the uncharacterized gene TGGT1_222100 (Figure 2B), which, based on the following results, we propose naming *Tg*SOS1. The pseudo-bulk transcriptome of sg(SOS1)-expressing cells correlated most strongly with the rhoptry effector kinase ROP16 (Figure 2D), which has been shown to directly phosphorylate STAT3 and STAT6 to activate downstream transcriptional programs.[43,44] Correspondingly, the most strongly downregulated genes in sg(SOS1)-expressing cells were CCL11 and CCL26, both of which have been shown to be regulated by STAT6[52,53] and were previously identified among the most strongly downregulated genes in *T. gondii* RHΔROP16-infected HFFs compared with RH wild type-infected HFFs[44] (Figure 4A; Table S9). Similarly, the most strongly downregulated pathway in these cells was the response to IL-4, which is the canonical activator of STAT6 signaling[54–57] (Figure 4B; Table S8). Interestingly, knockout of SOS1 appeared to significantly perturb more host cell pathways than knockout of ROP16 (Table S8). Although this may be an artifact of the screening methodology, it could also suggest that SOS1 has a broader function than ROP16.

SOS1 co-localizes with the rhoptry bulb marker ROP1 (Figure 4C). SOS1 has a predicted molecular weight of 128 kDa and appears to be cleaved approximately in the middle, with the tagged C-terminal fragment migrating at 70 kDa (Figure S9B). This processing could be inhibited with the small molecule 49c, an inhibitor of the Golgi-resident aspartyl protease ASP3[58] (Figure S9D). This result indicates that despite the apparent absence of a signal peptide, SOS1 is within the secretory pathway and contained within the lumen of the rhoptry bulb. This putative ASP3 cleavage site lies within a predicted flexible linker between two uncharacterized domains (Figure S9E). Unusually, for a rhoptry protein, the predicted C-terminal domain is conserved across Apicomplexa, whereas the N-terminal domain is recognizable only in Coccidia.

Based on the apparent similarity of sg(SOS1)-expressing cells to sg(ROP16)-expressing cells, we hypothesized that knockout of SOS1 would phenocopy knockout of ROP16 in loss of host STAT6 phosphorylation. We generated RHΔSOS1 and RHΔROP16 knockout cell lines, complemented RHΔSOS1 by inserting an HA-tagged copy of SOS1 (Figure S10), and measured phospho-STAT6 immunofluorescence in HFFs infected with these strains. Surprisingly, we did not find any difference in STAT6 phosphorylation at 1 hpi for RHΔSOS1 compared with RHΔKU80, despite observing the expected loss of this phosphorylation in cells infected with the RHΔROP16 cell line (Figure 4D). However, at 24 hpi, the same time point at which the dual perturb-seq data were collected, we found that STAT6 signaling was completely abolished in the RHΔSOS1-infected cells and rescued by complementation. Therefore, although ROP16 is sufficient for the initial phosphorylation of STAT6, SOS1 is required to sustain STAT6 signaling throughout the infection. Hence, we name this gene (TGGT1_222100) Sustainer Of STAT signaling 1.

Activation of STAT6 signaling by ROP16 has been shown to induce M2 polarization of *T. gondii*-infected macrophages.[59] Polarization is the process by which macrophages are activated to produce distinct functional phenotypes in response to specific stimuli; M2 polarization is canonically induced by the cytokines IL-4 and IL-13 and is associated with an anti-inflammatory phenotype.[60] During *T. gondii* infection *in vivo*, M2 polarization is implicated in dampening antiparasitic immune responses.[59,61] To test if SOS1-dependent sustained STAT6 phosphorylation is important for M2 polarization, we infected murine bone marrow-derived macrophages (BMDMs) and measured arginase activity, a defining feature of M2 polarization.[62] Arginase activity was strongly induced by infection with RHΔKU80 but was reduced to near the level in uninfected cells by knockout of either ROP16 or SOS1 (Figure 4E). These results show that the rhoptry kinase ROP16 is not sufficient for sustained host cell reprogramming and reveal a requirement for the previously unknown effector protein, SOS1. Although ROP16 is required for the initial phosphorylation of STAT6, SOS1 is necessary to sustain this signaling and thereby facilitate durable M2 polarization of infected macrophages (Figure 4F).

SOS1 maintains efficient bradyzoite cyst formation in neurons

During acute infection *in vivo*, a small percentage of *T. gondii* tachyzoites differentiate into bradyzoites, forming tissue cysts that are highly resistant to antiparasitic drugs and clearance by the host immune system. Bradyzoite cysts can persist for the lifetime of the host and are essential in the *T. gondii* life cycle for transmission to the definitive felid host[63]; moreover, they are clinically important as a source of recrudescent infection in immunocompromised individuals.[64] ROP16-dependent STAT6 signaling has previously been shown to facilitate bradyzoite cyst formation *in vivo* and *in vitro*, although the downstream host cell pathways involved remain unknown.[65,66] The findings here for SOS1 raised the question of whether sustained STAT6 signaling is necessary to maintain a permissive host cell environment for cyst formation or whether only the initial ROP16-dependent burst of signaling is necessary to trigger cyst formation.

As the *T. gondii* RH strain used in this work so far exhibits a very low rate of differentiation to bradyzoites,[67] to investigate the role of SOS1 in cyst formation, we generated a SOS1 knockout in the type III *T. gondii* CEP strain (Figure S11). As in the RH strain, we found that infection of HFFs with CEPΔSOS1 induced an initial ROP16-dependent burst of STAT6 signaling that dissipated after 24 h (Figure 5A). As *T. gondii* forms bradyzoite cysts primarily in neurons and skeletal muscle *in vivo*, we also measured STAT6 phosphorylation in infected primary murine neurons. We

CellPress
OPEN ACCESS

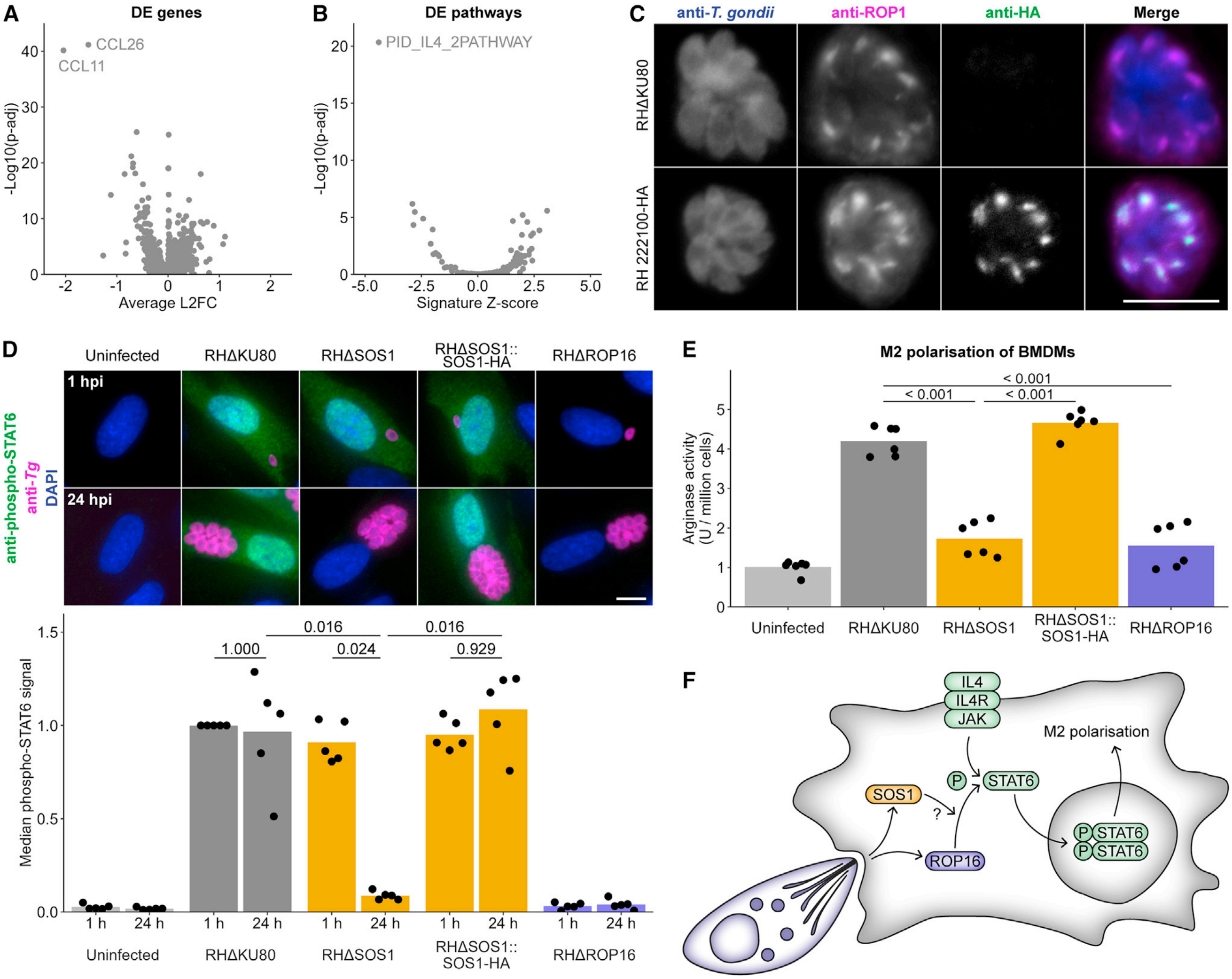

Figure 4. SOS1 (TGGT1_222100) is required for sustained STAT6 signaling in infected cells

(A) Differentially expressed host cell genes for sg(SOS1)-expression cells compared with all other (non-control) cells (two-sided Wilcoxon rank-sum test with Benjamini-Hochberg adjustment).

See also Table S9.

(B) Differentially expressed PID gene sets for sg(SOS1)-expression cells compared with all other (non-control) cells (two-sided Wilcoxon rank-sum test with Benjamini-Hochberg adjustment).

See also Table S8.

(C) Immunofluorescence localization of SOS1-HA in intracellular *T. gondii* parasites at 24 hpi. Scale bars, 10 μm.

(D) Quantification of nuclear phospho-STAT6 immunofluorescence in the nuclei of infected HFFs at 1 and 24 h post-infection. Scale bars, 10 μm. Differences tested by two-sided Wilcoxon rank-sum test with Bonferroni adjustment.

(E) Arginase activity of infected cell lysate. Differences tested by two-sided t test with Bonferroni adjustment.

(F) Model of ROP16- and SOS1-dependent regulation of STAT6 signaling.

observed the same loss of STAT6 phosphorylation at 24 hpi in neurons infected with CEPΔSOS1, compared with the parental strain, as we observed in HFFs, confirming that this phenotype is independent of host cell type (Figure 5B).

To determine whether SOS1 is required for efficient cyst formation, as is ROP16, we used high-content imaging and automated image analysis to quantify parasite differentiation from tachyzoites to bradyzoites in primary murine neurons.[66] Conversion of tachyzoite vacuoles to bradyzoite cysts was determined by staining of the cyst wall with *Dolichos bisflorus* agglutinin (DBA) (Figure 5C). At 24 hpi, there was no difference in the percentage of DBA-positive vacuoles between the parental CEP strain and CEPΔSOS1. However, at later time points, the rate of bradyzoite conversion in the CEPΔSOS1 line appeared to decrease until, at 72 hpi, the percentage of DBA-positive vacuoles was significantly reduced compared with the parental CEP stain and indistinguishable from CEPΔROP16. These findings mirror the dependence of sustained STAT6 signaling and M2 polarization on SOS1, showing that SOS1 is also critical to maintain efficient cyst formation (Figure 5D).

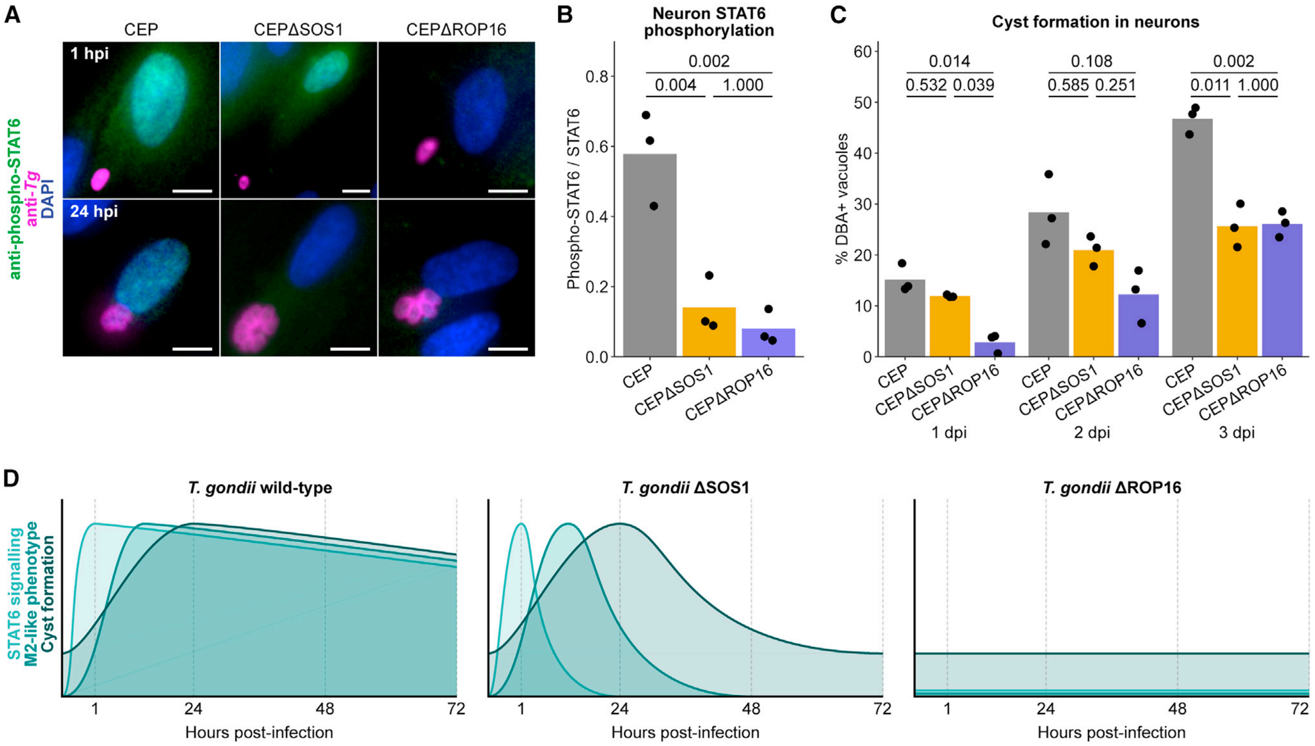

Figure 5. SOS1 maintains efficient bradyzoite cyst formation in neurons

(A) Phospho-STAT6 immunofluorescence in HFFs infected with the indicated *T. gondii* strains at 1 and 24 h post-infection. Scale bars, 10 μm.

(B) Quantification of phospho-STAT6 relative to total STAT6 from western blots of samples of primary murine neurons infected for 24 h with indicated *T. gondii* strains. Differences tested by two-sided t test with Bonferroni adjustment.

(C) Percentage of DBA-positive bradyzoite cysts out of total vacuoles with ≥ 2 parasites in primary murine neurons infected for 1, 2, or 3 days. Differences test by two-sided t test with Bonferroni adjustment.

(D) Model of the dynamics of ROP16- and SOS1-dependent phenotypes.

DISCUSSION

In this work, we combined pooled knockout screening with multi-modal scRNA-seq to overcome prior challenges in identifying *T. gondii* effector proteins that manipulate host cell gene expression. We propose this dual perturb-seq as a method to investigate transcriptional interactions in any host-pathogen or host-symbiont models that are amenable to genetic manipulation using CRISPR-Cas systems, with only minor modifications to the experimental strategy and analysis described here being necessary. For example, here, we rely on non-homologous end-joining to induce loss-of-function mutations, a pathway that is absent in many protozoan parasites. However, this requirement could be obviated by replacement of Cas9 in the perturb-seq vector with catalytically inactive Cas9, for CRISPRi, or Cas13, to target RNA transcripts. Furthermore, although sgRNAs are typically delivered by lentiviral transduction, we instead deliver both Cas9 and the sgRNA simultaneously by electroporation with a plasmid vector, highlighting the feasibility of perturb-seq in diverse non-mammalian cells.

While this paper was under review, a conceptually similar method for analyzing the impact of mutations in intracellular bacteria on the host cell transcriptome was published, which the authors named scPAIR-seq.[68] Wild-type and 24 previously generated mutant *Salmonella* strains[69] were engineered to express a polyadenylated GFP transcript containing a unique barcode to identify each mutant in pooled scRNA-seq data, in contrast to dual perturb-seq in which simultaneous gene disruption and barcoding is achieved by transfection with a Cas9-sgRNA plasmid library in a pooled format. scPAIR-seq does not recover the bacterial transcriptome, although prior demonstration of dual host-bacterium scRNA-seq of *Salmonella*-infected macrophages suggests that this may incorporated into scPAIR-seq in the future.[70] scPAIR-seq therefore currently offers less flexibility and throughput than dual perturb-seq yet will likely be an important complementary tool to dissect bacteria-host transcriptional interactions where dual perturb-seq is applicable for eukaryotic microbe-host interactions.

For *T. gondii*, a ubiquitous and clinically significant pathogen, we were able to use dual perturb-seq data to infer the functions of previously uncharacterized genes *de novo* and in relation to established effector proteins. Importantly, despite critical roles in infection *in vivo*, effector proteins that target host cell transcription have not been identified by growth competition-based knockout screens *in vitro* and *in vivo*.[7–10] This is likely because the primary role of these effectors in modulating host immunity is rescued in a pooled infection.[7] This phenomenon necessitated the use of dual perturb-seq to directly measure the impact of *T. gondii* gene knockout on the host cell transcriptome in a high-throughput and unbiased manner.

We validated the dual perturb-seq phenotype of GRA59 by showing that it contributes to export of other dense granule effectors into the host cell. Based on these phenotypes, plus the localization and topology of GRA59, we suggest that it may be an accessory or regulatory component of the export complex, facilitating efficient effector export into the host cell. Interestingly, we do not identify any mutants that completely ablate effector export other than the core cluster comprising MYR1–4, ROP17, and GRA45. Having thus defined all components required for protein export paves the way for detailed mechanistic and structural studies of this pathway, which is currently poorly understood yet critical for *T. gondii* virulence.[5,15]

Additionally, we identify an effector protein, SOS1, which operates via a previously unrecognized mode of action to prolong STAT6 signaling initiated by the ROP16 kinase. Such a finding was unexpected, as recombinant ROP16 is sufficient to phosphorylate STAT6 *in vitro*.[43,44] In a cellular context, SOS1 may regulate the activity or stability of ROP16 or may disrupt feedback mechanisms that downregulate STAT6 activity. We show here that sustained STAT6 signaling is necessary for M2 polarization of *T. gondii*-infected macrophages and efficient bradyzoite cyst formation in neurons. Through these phenotypes, SOS1 is implicated in parasite immune evasion and transmission *in vivo*.

With dual perturb-seq, we are thus able to identify effector proteins in a high-throughput and systematic manner and uncover mechanisms of effector-mediated host cell reprogramming. Dual perturb-seq will be a powerful tool to accelerate the study of *T. gondii* effector proteins across different strains and host cell types and to identify the molecular mediators of transcriptional remodeling in other host-microbe interactions.

STAR★METHODS

Detailed methods are provided in the online version of this paper and include the following:

- KEY RESOURCES TABLE
- RESOURCE AVAILABILITY
  - Lead contact
  - Materials availability
  - Data and code availability
- EXPERIMENTAL MODEL AND SUBJECT DETAILS
  - Mice
  - Cell culture
- METHOD DETAILS
  - Generation of perturb-seq vectors
  - Dual perturb-seq experiments
  - Generation of *T. gondii* cell lines
  - Western blot
  - Plaque assay
  - Immunofluorescence assays
  - Arginase activity assay
  - Identification of protein homologues
  - *In vitro* cyst formation assay
- QUANTIFICATION AND STATISTICAL ANALYSIS
  - Analysis of dual perturb-seq data

SUPPLEMENTAL INFORMATION

Supplemental information can be found online at https://doi.org/10.1016/j.chom.2023.09.003.

ACKNOWLEDGMENTS

We thank the Cell Services, High Throughput Screening, Flow Cytometry, and Advanced Sequencing Facility Science Technology Platforms at the Francis Crick Institute for support. We thank the laboratory of Dominique Soldati for providing 49c. We thank Manfred Claassen and Revant Gupta for discussion and advice on data analysis. We thank VEuPathDB for providing access to genomic and other large-scale datasets for *T. gondii*. This work was supported by an award to M.T. from the Wellcome Trust (223192/Z/21/Z), by funding to M.T. from the Francis Crick Institute, which receives its core funding from Cancer Research UK (CC2132), the UK Medical Research Council (CC2132), and the Wellcome Trust (CC2132), by funding to A.A.K. from the College of Medicine, University of Arizona, and by funding to A.A.K. from the BIO5 Institute, University of Arizona. F.T. is funded by the Deutsche Forschungsgemeinschaft (TO 1349/1-1). The Science Technology Platforms at the Francis Crick Institute receive funding from Cancer Research UK (CC0199), the UK Medical Research Council (CC0199), and the Wellcome Trust (CC0199). For the purpose of open access, the authors have applied a CC BY public copyright license to any Author Accepted Manuscript version arising from this submission.

AUTHOR CONTRIBUTIONS

Conceptualization, S.B. and M.T.; methodology, S.B. and A.E.; investigation, S.B., S.C., K.K.T., F.T., and E.J.L.; formal analysis, S.B. and K.K.; visualization, S.B.; writing – original draft, S.B. and M.T.; writing – review and editing, all authors; supervision, R.G., A.A.K., and M.T.; funding acquisition, A.A.K. and M.T.

DECLARATION OF INTERESTS

The authors declare no competing interests.

Received: June 5, 2023
Revised: August 4, 2023
Accepted: September 7, 2023
Published: October 11, 2023

REFERENCES

1. Keeling, P.J., and McCutcheon, J.P. (2017). Endosymbiosis: the feeling is not mutual. J. Theor. Biol. *434*, 75–79.
2. Jacobovitz, M.R., Rupp, S., Voss, P.A., Maegele, I., Gornik, S.G., and Guse, A. (2021). Dinoflagellate symbionts escape vomocytosis by host cell immune suppression. Nat. Microbiol. *6*, 769–782.
3. Hakimi, M.-A., Olias, P., and Sibley, L.D. (2017). *Toxoplasma* effectors targeting Host Signaling and Transcription. Clin. Microbiol. Rev. *30*, 615–645. https://doi.org/10.1128/CMR.00005-17.
4. Hanford, H.E., Von Dwingelo, J., and Abu Kwaik, Y. (2021). Bacterial nucleomodulins: A coevolutionary adaptation to the eukaryotic command center. PLoS Pathog. *17*, e1009184.
5. Rastogi, S., Cygan, A.M., and Boothroyd, J.C. (2019). Translocation of effector proteins into host cells by Toxoplasma gondii. Curr. Opin. Microbiol. *52*, 130–138.
6. Barylyuk, K., Koreny, L., Ke, H., Butterworth, S., Crook, O.M., Lassadi, I., Gupta, V., Tromer, E., Mourier, T., Stevens, T.J., et al. (2020). A comprehensive subcellular atlas of the toxoplasma proteome via hyperLOPIT provides spatial context for protein functions. Cell Host Microbe *28*, 752–766.e9.
7. Young, J., Dominicus, C., Wagener, J., Butterworth, S., Ye, X., Kelly, G., Ordan, M., Saunders, B., Instrell, R., Howell, M., et al. (2019). A CRISPR platform for targeted in vivo screens identifies Toxoplasma gondii virulence factors in mice. Nat. Commun. *10*, 3963.

8. Sangaré, L.O., Ólafsson, E.B., Wang, Y., Yang, N., Julien, L., Camejo, A., Pesavento, P., Sidik, S.M., Lourido, S., Barragan, A., et al. (2019). In vivo CRISPR screen identifies TgWIP as a toxoplasma modulator of dendritic cell migration. Cell Host Microbe *26*, 478–492.e8.

9. Wang, Y., Sangaré, L.O., Paredes-Santos, T.C., Hassan, M.A., Krishnamurthy, S., Furuta, A.M., Markus, B.M., Lourido, S., and Saeij, J.P.J. (2020). Genome-wide screens identify Toxoplasma gondii determinants of parasite fitness in IFNγ-activated murine macrophages. Nat. Commun. *11*, 5258.

10. Butterworth, S., Torelli, F., Lockyer, E.J., Wagener, J., Song, O.-R., Broncel, M., Russell, M.R.G., Moreira-Souza, A.C.A., Young, J.C., and Treeck, M. (2022). Toxoplasma gondii virulence factor ROP1 reduces parasite susceptibility to murine and human innate immune restriction. PLoS Pathog. *18*, e1011021.

11. Replogle, J.M., Norman, T.M., Xu, A., Hussmann, J.A., Chen, J., Cogan, J.Z., Meer, E.J., Terry, J.M., Riordan, D.P., Srinivas, N., et al. (2020). Combinatorial single-cell CRISPR screens by direct guide RNA capture and targeted sequencing. Nat. Biotechnol. *38*, 954–961.

12. Donald, R.G., and Roos, D.S. (1995). Insertional mutagenesis and marker rescue in a protozoan parasite: cloning of the uracil phosphoribosyltransferase locus from Toxoplasma gondii. Proc. Natl. Acad. Sci. USA *92*, 5749–5753.

13. Behnke, M.S., Wootton, J.C., Lehmann, M.M., Radke, J.B., Lucas, O., Nawas, J., Sibley, L.D., and White, M.W. (2010). Coordinated progression through two subtranscriptomes underlies the tachyzoite cycle of Toxoplasma gondii. PLoS One *5*, e12354.

14. Radke, J.R., Striepen, B., Guerini, M.N., Jerome, M.E., Roos, D.S., and White, M.W. (2001). Defining the cell cycle for the tachyzoite stage of Toxoplasma gondii. Mol. Biochem. Parasitol. *115*, 165–175.

15. Franco, M., Panas, M.W., Marino, N.D., Lee, M.-C.W., Buchholz, K.R., Kelly, F.D., Bednarski, J.J., Sleckman, B.P., Pourmand, N., and Boothroyd, J.C. (2016). A Novel Secreted Protein, MYR1, Is Central to Toxoplasma's Manipulation of Host Cells. mBio *7*. e02231–e02215.

16. Naor, A., Panas, M.W., Marino, N., Coffey, M.J., Tonkin, C.J., and Boothroyd, J.C. (2018). MYR1-dependent effectors are the major drivers of a host cell's early response to Toxoplasma, including counteracting MYR1-independent effects. mBio *9*. e02401–e02417. https://doi.org/10.1128/mBio.02401-17.

17. Panas, M.W., Naor, A., Cygan, A.M., and Boothroyd, J.C. (2019). Toxoplasma controls Host Cyclin E Expression through the Use of a Novel MYR1-Dependent Effector Protein, HCE1. mBio *10*. e00674–e00619. https://doi.org/10.1128/mBio.00674-19.

18. Braun, L., Brenier-Pinchart, M.-P., Hammoudi, P.-M., Cannella, D., Kieffer-Jaquinod, S., Vollaire, J., Josserand, V., Touquet, B., Couté, Y., Tardieux, I., et al. (2019). The Toxoplasma effector TEEGR promotes parasite persistence by modulating NF-κB signalling via EZH2. Nat. Microbiol. *4*, 1208–1220.

19. Olias, P., Etheridge, R.D., Zhang, Y., Holtzman, M.J., and Sibley, L.D. (2016). Toxoplasma effector recruits the Mi-2/NuRD complex to repress STAT1 transcription and block IFN-γ-dependent gene expression. Cell Host Microbe *20*, 72–82.

20. Gay, G., Braun, L., Brenier-Pinchart, M.-P., Vollaire, J., Josserand, V., Bertini, R.-L., Varesano, A., Touquet, B., De Bock, P.-J., Coute, Y., et al. (2016). Toxoplasma gondii TgIST co-opts host chromatin repressors dampening STAT1-dependent gene regulation and IFN-γ-mediated host defenses. J. Exp. Med. *213*, 1779–1798.

21. Bougdour, A., Durandau, E., Brenier-Pinchart, M.-P., Ortet, P., Barakat, M., Kieffer, S., Curt-Varesano, A., Curt-Bertini, R.-L., Bastien, O., Coute, Y., et al. (2013). Host cell subversion by Toxoplasma GRA16, an exported dense granule protein that targets the host cell nucleus and alters gene expression. Cell Host Microbe *13*, 489–500.

22. Braun, L., Brenier-Pinchart, M.-P., Yogavel, M., Curt-Varesano, A., Curt-Bertini, R.-L., Hussain, T., Kieffer-Jaquinod, S., Coute, Y., Pelloux, H., Tardieux, I., et al. (2013). A Toxoplasma dense granule protein, GRA24, modulates the early immune response to infection by promoting a direct and sustained host p38 MAPK activation. J. Exp. Med. *210*, 2071–2086.

23. Rudzki, E.N., Ander, S.E., Coombs, R.S., Alrubaye, H.S., Cabo, L.F., Blank, M.L., Gutiérrez-Melo, N., Dubey, J.P., Coyne, C.B., and Boyle, J.P. (2021). Toxoplasma gondii GRA28 is required for placenta-specific induction of the regulatory chemokine CCL22 in human and mouse. mBio *12*, e0159121.

24. Ten Hoeve, A.L., Braun, L., Rodriguez, M.E., Olivera, G.C., Bougdour, A., Belmudes, L., Couté, Y., Saeij, J.P.J., Hakimi, M.-A., and Barragan, A. (2022). The Toxoplasma effector GRA28 promotes parasite dissemination by inducing dendritic cell-like migratory properties in infected macrophages. Cell Host Microbe *30*, 1570–1588.e7.

25. Saeij, J.P.J., Coller, S., Boyle, J.P., Jerome, M.E., White, M.W., and Boothroyd, J.C. (2007). Toxoplasma co-opts host gene expression by injection of a polymorphic kinase homologue. Nature *445*, 324–327.

26. Ma, J.S., Sasai, M., Ohshima, J., Lee, Y., Bando, H., Takeda, K., and Yamamoto, M. (2014). Selective and strain-specific NFAT4 activation by the Toxoplasma gondii polymorphic dense granule protein GRA6. J. Exp. Med. *211*, 2013–2032.

27. Yang, C.-S., Yuk, J.-M., Lee, Y.-H., and Jo, E.-K. (2016). Toxoplasma gondii GRA7-induced TRAF6 activation contributes to host protective immunity. Infect. Immun. *84*, 339–350.

28. Li, J.-X., He, J.-J., Elsheikha, H.M., Ma, J., Xu, X.-P., and Zhu, X.-Q. (2020). ROP18-mediated transcriptional reprogramming of HEK293T cell reveals new roles of ROP18 in the interplay between Toxoplasma gondii and the host cell. Front. Cell. Infect. Microbiol. *10*, 586946.

29. Rosowski, E.E., Lu, D., Julien, L., Rodda, L., Gaiser, R.A., Jensen, K.D.C., and Saeij, J.P.J. (2011). Strain-specific activation of the NF-kappaB pathway by GRA15, a novel Toxoplasma gondii dense granule protein. J. Exp. Med. *208*, 195–212.

30. He, H., Brenier-Pinchart, M.-P., Braun, L., Kraut, A., Touquet, B., Couté, Y., Tardieux, I., Hakimi, M.-A., and Bougdour, A. (2018). Characterization of a Toxoplasma effector uncovers an alternative GSK3/β-catenin-regulatory pathway of inflammation. eLife *7*, e39887. https://doi.org/10.7554/eLife.39887.

31. Shastri, A.J., Marino, N.D., Franco, M., Lodoen, M.B., and Boothroyd, J.C. (2014). GRA25 is a novel virulence factor of Toxoplasma gondii and influences the host immune response. Infect. Immun. *82*, 2595–2605.

32. DeTomaso, D., Jones, M.G., Subramaniam, M., Ashuach, T., Ye, C.J., and Yosef, N. (2019). Functional interpretation of single cell similarity maps. Nat. Commun. *10*, 4376.

33. Amos, B., Aurrecoechea, C., Barba, M., Barreto, A., Basenko, E.Y., Bażant, W., Belnap, R., Blevins, A.S., Böhme, U., Brestelli, J., et al. (2022). VEuPathDB: the eukaryotic pathogen, vector and host bioinformatics resource center. Nucleic Acids Res. *50*, D898–D911.

34. Rosenberg, A., and Sibley, L.D. (2021). Toxoplasma gondii secreted effectors co-opt host repressor complexes to inhibit necroptosis. Cell Host Microbe *29*, 1186–1198.e8.

35. Sidik, S.M., Huet, D., Ganesan, S.M., Huynh, M.-H., Wang, T., Nasamu, A.S., Thiru, P., Saeij, J.P.J., Carruthers, V.B., Niles, J.C., et al. (2016). A genome-wide CRISPR screen in toxoplasma identifies essential apicomplexan. Genes Cells *166*, 1423–1435.e12.

36. Hotelling, H. (1931). The generalization of Student's ratio. Ann. Math. Statist. *2*, 360–378.

37. Mayoral, J., Tomita, T., Tu, V., Aguilan, J.T., Sidoli, S., and Weiss, L.M. (2020). Toxoplasma gondii PPM3C, a secreted protein phosphatase, affects parasitophorous vacuole effector export. PLoS Pathog. *16*, e1008771.

38. Shen, B., Brown, K.M., Lee, T.D., and Sibley, L.D. (2014). Efficient gene disruption in diverse strains of Toxoplasma gondii using CRISPR/CAS9. mBio *5*. e01114–e01114. https://doi.org/10.1128/mBio.01114-14.

39. Marino, N.D., Panas, M.W., Franco, M., Theisen, T.C., Naor, A., Rastogi, S., Buchholz, K.R., Lorenzi, H.A., and Boothroyd, J.C. (2018). Identification of a novel protein complex essential for effector

CellPress
OPEN ACCESS

translocation across the parasitophorous vacuole membrane of Toxoplasma gondii. PLoS Pathog. *14*, e1006828.

40. Cygan, A.M., Theisen, T.C., Mendoza, A.G., Marino, N.D., Panas, M.W., and Boothroyd, J.C. (2020). Coimmunoprecipitation with MYR1 identifies three additional proteins within the Toxoplasma gondii parasitophorous vacuole required for translocation of dense granule effectors into host cells. mSphere *5*. e00858–e00819. https://doi.org/10.1128/mSphere.00858-19.

41. Panas, M.W., Ferrel, A., Naor, A., Tenborg, E., Lorenzi, H.A., and Boothroyd, J.C. (2019). Translocation of dense granule effectors across the parasitophorous vacuole membrane in Toxoplasma- infected cells requires the activity of ROP17, a rhoptry protein kinase. mSphere *4*. e00276–e00219. https://doi.org/10.1128/mSphere.00276-19.

42. Blakely, W.J., Holmes, M.J., and Arrizabalaga, G. (2020). The secreted acid phosphatase domain-containing GRA44 from Toxoplasma gondii is required for c-Myc induction in infected cells. mSphere *5*. e00877–e00819. https://doi.org/10.1128/mSphere.00877-19.

43. Yamamoto, M., Standley, D.M., Takashima, S., Saiga, H., Okuyama, M., Kayama, H., Kubo, E., Ito, H., Takaura, M., Matsuda, T., et al. (2009). A single polymorphic amino acid on Toxoplasma gondii kinase ROP16 determines the direct and strain-specific activation of Stat3. J. Exp. Med. *206*, 2747–2760.

44. Ong, Y.-C., Reese, M.L., and Boothroyd, J.C. (2010). Toxoplasma rhoptry protein 16 (ROP16) subverts host function by direct tyrosine phosphorylation of STAT6. J. Biol. Chem. *285*, 28731–28740.

45. Schaefer, C.F., Anthony, K., Krupa, S., Buchoff, J., Day, M., Hannay, T., and Buetow, K.H. (2009). PID: the pathway interaction database. Nucleic Acids Res. *37*, D674–D679.

46. Liberzon, A., Subramanian, A., Pinchback, R., Thorvaldsdóttir, H., Tamayo, P., and Mesirov, J.P. (2011). Molecular signatures database (MSigDB) 3.0. Bioinformatics *27*, 1739–1740.

47. Panas, M.W., and Boothroyd, J.C. (2020). *Toxoplasma* Uses GRA16 to upregulate Host c-Myc. mSphere *5*. e00402–e00420. https://doi.org/10.1128/mSphere.00402-20.

48. Nadipuram, S.M., Kim, E.W., Vashisht, A.A., Lin, A.H., Bell, H.N., Coppens, I., Wohlschlegel, J.A., and Bradley, P.J. (2016). In vivo biotinylation of the toxoplasma parasitophorous vacuole reveals novel dense granule proteins important for parasite growth and pathogenesis. mBio *7*. e00808–e00816. https://doi.org/10.1128/mBio.00808-16.

49. Cygan, A.M., Jean Beltran, P.M., Mendoza, A.G., Branon, T.C., Ting, A.Y., Carr, S.A., and Boothroyd, J.C. (2021). Proximity-labeling reveals novel Host and Parasite Proteins at the Toxoplasma Parasitophorous Vacuole Membrane. mBio *12*, e0026021.

50. Lockyer, E.J., Torelli, F., Butterworth, S., Song, O.-R., Howell, S., Weston, A., East, P., and Treeck, M. (2023). A heterotrimeric complex of Toxoplasma proteins promotes parasite survival in interferon gamma-stimulated human cells. PLoS Biol. *21*, e3002202.

51. Krishnamurthy, S., Maru, P., Wang, Y., Bitew, M.A., Mukhopadhyay, D., Yamaryo-Botté, Y., Paredes-Santos, T.C., Sangaré, L.O., Swale, C., Botté, C.Y., et al. (2023). CRISPR screens identify Toxoplasma genes that determine parasite fitness in interferon gamma-stimulated human cells. mBio *14*, e0006023.

52. Matsukura, S., Stellato, C., Plitt, J.R., Bickel, C., Miura, K., Georas, S.N., Casolaro, V., and Schleimer, R.P. (1999). Activation of eotaxin gene transcription by NF-kappa B and STAT6 in human airway epithelial cells. J. Immunol. *163*, 6876–6883.

53. Hoeck, J., and Woisetschläger, M. (2001). Activation of eotaxin-3/CCLl26 gene expression in human dermal fibroblasts is mediated by STAT6. J. Immunol. *167*, 3216–3222.

54. Kotanides, H., and Reich, N.C. (1993). Requirement of tyrosine phosphorylation for rapid activation of a DNA binding factor by IL-4. Science *262*, 1265–1267. https://doi.org/10.1126/science.7694370.

55. Hou, J., Schindler, U., Henzel, W.J., Ho, T.C., Brasseur, M., and McKnight, S.L. (1994). An interleukin-4-induced transcription factor: il-4 stat. Science *265*, 1701–1706.

56. Schindler, C., Kashleva, H., Pernis, A., Pine, R., and Rothman, P. (1994). STF-IL-4: a novel IL-4-induced signal transducing factor. EMBO J. *13*, 1350–1356. https://doi.org/10.1002/j.1460-2075.1994.tb06388.x.

57. Quelle, F.W., Shimoda, K., Thierfelder, W., Fischer, C., Kim, A., Ruben, S.M., Cleveland, J.L., Pierce, J.H., Keegan, A.D., and Nelms, K. (1995). Cloning of murine Stat6 and human Stat6, Stat proteins that are tyrosine phosphorylated in responses to IL-4 and IL-3 but are not required for mitogenesis. Mol. Cell. Biol. *15*, 3336–3343.

58. Dogga, S.K., Mukherjee, B., Jacot, D., Kockmann, T., Molino, L., Hammoudi, P.-M., Hartkoorn, R.C., Hehl, A.B., and Soldati-Favre, D. (2017). A druggable secretory protein maturase of Toxoplasma essential for invasion and egress. eLife *6*, e27480. https://doi.org/10.7554/eLife.27480.

59. Jensen, K.D.C., Wang, Y., Wojno, E.D.T., Shastri, A.J., Hu, K., Cornel, L., Boedec, E., Ong, Y.-C., Chien, Y.-H., Hunter, C.A., et al. (2011). Toxoplasma polymorphic effectors determine macrophage polarization and intestinal inflammation. Cell Host Microbe *9*, 472–483.

60. Murray, P.J., and Wynn, T.A. (2011). Protective and pathogenic functions of macrophage subsets. Nat. Rev. Immunol. *11*, 723–737.

61. Chen, L., Christian, D.A., Kochanowsky, J.A., Phan, A.T., Clark, J.T., Wang, S., Berry, C., Oh, J., Chen, X., Roos, D.S., et al. (2020). The Toxoplasma gondii virulence factor ROP16 acts in cis and trans, and suppresses T cell responses. J. Exp. Med. *217*, e20181757. https://doi.org/10.1084/jem.20181757.

62. Mills, C.D., Kincaid, K., Alt, J.M., Heilman, M.J., and Hill, A.M. (2000). M-1/M-2 macrophages and the Th1/Th2 paradigm. J. Immunol. *164*, 6166–6173.

63. Dubey, J.P. (2014). Chapter 1 The history and life cycle of *Toxoplasma gondii*. In Toxoplasma gondii, Second Edition, L.M. Weiss and K. Kim, eds. (Academic Press), pp. 1–17.

64. Porter, S.B., and Sande, M.A. (1992). Toxoplasmosis of the central nervous system in the acquired immunodeficiency syndrome. N. Engl. J. Med. *327*, 1643–1648.

65. Tuladhar, S., Kochanowsky, J.A., Bhaskara, A., Ghotmi, Y., Chandrasekaran, S., and Koshy, A.A. (2019). The ROP16III-dependent early immune response determines the subacute CNS immune response and type III Toxoplasma gondii survival. PLoS Pathog. *15*, e1007856.

66. Kochanowsky, J.A., Chandrasekaran, S., Sanchez, J.R., Thomas, K.K., and Koshy, A.A. (2023). ROP16-mediated activation of STAT6 enhances cyst development of type III Toxoplasma gondii in neurons. PLoS Pathog. *19*, e1011347.

67. Weiss, L.M., and Kim, K. (2000). The development and biology of bradyzoites of Toxoplasma gondii. Front. Biosci. *5*, D391–D405.

68. Heyman, O., Yehezkel, D., Ciolli Mattioli, C., Blumberger, N., Rosenberg, G., Solomon, A., Hoffman, D., Bossel Ben-Moshe, N., and Avraham, R. (2023). Paired single-cell host profiling with multiplex-tagged bacterial mutants reveals intracellular virulence-immune networks. Proc. Natl. Acad. Sci. USA *120*, e2218812120.

69. Porwollik, S., Santiviago, C.A., Cheng, P., Long, F., Desai, P., Fredlund, J., Srikumar, S., Silva, C.A., Chu, W., Chen, X., et al. (2014). Defined single-gene and multi-gene deletion mutant collections in Salmonella enterica sv Typhimurium. PLoS One *9*, e99820.

70. Avital, G., Avraham, R., Fan, A., Hashimshony, T., Hung, D.T., and Yanai, I. (2017). scDual-Seq: mapping the gene regulatory program of Salmonella infection by host and pathogen single-cell RNA-sequencing. Genome Biol. *18*, 200.

71. Donald, R.G., Carter, D., Ullman, B., and Roos, D.S. (1996). Insertional tagging, cloning, and expression of the Toxoplasma gondii hypoxanthine-xanthine-guanine phosphoribosyltransferase gene. Use as a selectable marker for stable transformation. J. Biol. Chem. *271*, 14010–14019.

72. Huynh, M.-H., and Carruthers, V.B. (2009). Tagging of endogenous genes in a Toxoplasma gondii strain lacking Ku80. Eukaryot. Cell *8*, 530–539.

73. Schneider, C.A., Rasband, W.S., and Eliceiri, K.W. (2012). NIH Image to ImageJ: 25 years of image analysis. Nat. Methods *9*, 671–675.

74. Hafemeister, C., and Satija, R. (2019). Normalization and variance stabilization of single-cell RNA-seq data using regularized negative binomial regression. Genome Biol. *20*, 296.

75. Choudhary, S., and Satija, R. (2022). Comparison and evaluation of statistical error models for scRNA-seq. Genome Biol. *23*, 27.

76. Madisen, L., Zwingman, T.A., Sunkin, S.M., Oh, S.W., Zariwala, H.A., Gu, H., Ng, L.L., Palmiter, R.D., Hawrylycz, M.J., Jones, A.R., et al. (2010). A robust and high-throughput Cre reporting and characterization system for the whole mouse brain. Nat. Neurosci. *13*, 133–140.

77. Chandrasekaran, S., Kochanowsky, J.A., Merritt, E.F., Lagas, J.S., Swannigan, A., and Koshy, A.A. (2022). IFN-γ stimulated murine and human neurons mount anti-parasitic defenses against the intracellular parasite Toxoplasma gondii. Nat. Commun. *13*, 4605.

78. Su, C., Zhang, X., and Dubey, J.P. (2006). Genotyping of Toxoplasma gondii by multilocus PCR-RFLP markers: a high resolution and simple method for identification of parasites. Int. J. Parasitol. *36*, 841–848.

79. Shen, B., Brown, K., Long, S., and Sibley, L.D. (2017). Development of CRISPR/Cas9 for efficient genome editing in Toxoplasma gondii. Methods Mol. Biol. *1498*, 79–103.

80. Cabral, C.M., McGovern, K.E., MacDonald, W.R., Franco, J., and Koshy, A.A. (2017). Dissecting amyloid beta deposition using distinct strains of the neurotropic parasite *Toxoplasma gondii* as a novel tool. ASN Neuro *9*, 1759091417724915.

81. Jumper, J., Evans, R., Pritzel, A., Green, T., Figurnov, M., Ronneberger, O., Tunyasuvunakool, K., Bates, R., Žídek, A., Potapenko, A., et al. (2021). Highly accurate protein structure prediction with AlphaFold. Nature *596*, 583–589.

82. Varadi, M., Anyango, S., Deshpande, M., Nair, S., Natassia, C., Yordanova, G., Yuan, D., Stroe, O., Wood, G., Laydon, A., et al. (2022). AlphaFold Protein Structure Database: massively expanding the structural coverage of protein-sequence space with high-accuracy models. Nucleic Acids Res. *50*, D439–D444.

83. Potter, S.C., Luciani, A., Eddy, S.R., Park, Y., Lopez, R., and Finn, R.D. (2018). HMMER web server: 2018 update. Nucleic Acids Res. *46*, W200–W204.

84. Madeira, F., Pearce, M., Tivey, A.R.N., Basutkar, P., Lee, J., Edbali, O., Madhusoodanan, N., Kolesnikov, A., and Lopez, R. (2022). Search and sequence analysis tools services from EMBL-EBI in 2022. Nucleic Acids Res. *50*, W276–W279.

85. Hao, Y., Hao, S., Andersen-Nissen, E., Mauck, W.M., 3rd, Zheng, S., Butler, A., Lee, M.J., Wilk, A.J., Darby, C., Zager, M., et al. (2021). Integrated analysis of multimodal single-cell data. Cell *184*, 3573–3587.e29.

86. Ilicic, T., Kim, J.K., Kolodziejczyk, A.A., Bagger, F.O., McCarthy, D.J., Marioni, J.C., and Teichmann, S.A. (2016). Classification of low quality cells from single-cell RNA-seq data. Genome Biol. *17*, 29.

87. Matta, S.K., Olias, P., Huang, Z., Wang, Q., Park, E., Yokoyama, W.M., and Sibley, L.D. (2019). Toxoplasma gondii effector TgIST blocks type I interferon signaling to promote infection. Proc. Natl. Acad. Sci. USA *116*, 17480–17491.

STAR★METHODS

KEY RESOURCES TABLE

REAGENT or RESOURCE	SOURCE	IDENTIFIER
Antibodies		
Goat polyclonal anti-mouse Alexa Fluor 488 conjugate	ThermoFisher Scientific	Cat#A-11001; RRID: AB_2534069
Goat polyclonal anti-mouse Alexa Fluor 647 conjugate	ThermoFisher Scientific	Cat#A-21235; RRID: AB_2535804
Goat polyclonal anti-mouse IRDye 680LT conjugate	LI-COR Biosciences	Cat#925-68020; RRID: AB_2687826
Goat polyclonal anti-rabbit Alexa Fluor 488 conjugate	ThermoFisher Scientific	Cat#A-11008; RRID: AB_143165
Goat polyclonal anti-rabbit Alexa Fluor 647 conjugate	ThermoFisher Scientific	Cat#A-21244; RRID: AB_2535812
Goat polyclonal anti-rat Alexa Fluor 594 conjugate	ThermoFisher Scientific	Cat#A-11007; RRID: AB_10561522
Goat polyclonal anti-rat IRDye 800CW conjugate	LI-COR Biosciences	Cat#925-32219; RRID: AB_2721932
Mouse monoclonal anti-ROP1	Abnova	Cat#MAB17504
Mouse monoclonal anti-*T. gondii*	Santa Cruz Biotechnology	Cat#sc-52255; RRID: AB_630350
Mouse monoclonal anti-EZH2	BD	Cat#612666; RRID: AB_2102429
Mouse monoclonal anti-β-actin	Cell Signaling Technology	Cat#3700; RRID: AB_2242334
Mouse polyclonal anti-GRA1	Laboratory of Jean-Francois Dubremetz	N/A
Mouse polyclonal anti-GRA2	Laboratory of Jean-Francois Dubremetz	N/A
Mouse polyclonal anti-SAG1	Laboratory of John Boothroyd	N/A
Rabbit monoclonal anti-cMyc	Cell Signaling Technology	Cat#5605; RRID: AB_1903938
Rabbit monoclonal anti-IRF1	Cell Signaling Technology	Cat#8478; RRID: AB_10949108
Rabbit monoclonal anti-phospho-p38	Cell Signaling Technology	Cat#4511; RRID: AB_2139682
Rabbit monoclonal anti-phospho-STAT6	Cell Signaling Technology	Cat#56554; RRID: AB_2799514
Rabbit monoclonal anti-STAT6	Cell Signaling Technology	Cat#5397; RRID: AB_11220421
Rabbit polyclonal anti-GRA3	Laboratory of Jean-Francois Dubremetz	N/A
Rabbit polyclonal anti-*T. gondii*	Abcam	Cat#ab138698
Rabbit polyclonal anti-*T. gondii*	ThermoFisher Scientific	Cat#PA1-7252; RRID: AB_561769
Rat monoclonal anti-HA	Sigma-Aldrich	Cat#11867423001; RRID: AB_390918
Chemicals, peptides, and recombinant proteins		
16% Formaldehyde (w/v), methanol-free	ThermoFisher Scientific	Cat#28908
2-mercaptoethanol	Sigma-Aldrich	Cat#M3701
5-Fluoro-2′-deoxyuridine	Sigma-Aldrich	Cat#F0503
AgeI-HF	NEB	Cat#R3552L
Ammonium oxalate	Sigma-Aldrich	Cat#221716
B-27 Supplement	ThermoFisher Scientific	Cat#17504001
Benzonase nuclease	Sigma-Aldrich	Cat#E1014
Bovine serum albumin	Sigma-Aldrich	Cat#A9647
cOmplete, Mini, EDTA-free Protease Inhibitor Cocktail	Sigma-Aldrich	Cat#11836170001
Crystal violet	Sigma-Aldrich	Cat#C6158
Disuccinimidyl glutarate	ThermoFisher Scientific	Cat#20593
DMEM (Dulbecco's Modified Eagle's Medium), high glucose, GlutaMAX™ Supplement	ThermoFisher Scientific	Cat#10566016

(*Continued on next page*)

Continued

REAGENT or RESOURCE	SOURCE	IDENTIFIER
DMEM, high glucose, no glutamine, no phenol red	ThermoFisher Scientific	Cat#31053028
Dolichos bisflorus agglutinin (DBA), biotinylated	Vector Laboratories	Cat#B-1035-5
Dulbecco's Phosphate Buffered Saline	Sigma-Aldrich	Cat#D8537
Fetal Bovine Serum, qualified, heat inactivated, Brazil	ThermoFisher Scientific	Cat#10500064
Fluoromount-G Mounting Medium	ThermoFisher Scientific	Cat#00-4958-02
Glycine	Sigma-Aldrich	Cat#50046
KpnI-HF	NEB	Cat#R3142L
L929 cell-conditioned medium	Cell Services Science Technology Platform, Francis Crick Institute	N/A
MEM (Minimal Essential Medium)	ThermoFisher Scientific	Cat#11095080
Mycophenolic acid	Sigma-Aldrich	Cat#475913
NciI	NEB	Cat#R0196L
Neurobasal Medium	ThermoFisher Scientific	Cat#21103049
Penicillin-Streptomycin-Glutamine (100X)	ThermoFisher Scientific	Cat#10378016
Phosphatase Inhibitor Cocktail 2	Sigma-Aldrich	Cat#P5726
Pierce anti-HA agarose	ThermoFisher Scientific	Cat#26181
Pierce RIPA buffer	ThermoFisher Scientific	Cat#89900
Protease Inhibitor Cocktail	Sigma-Aldrich	Cat#P8340
Recombinant Human IFN-gamma Protein	Bio-Techne	Cat#285-IF-100/CF
RPMI 1640 Medium (ATCC modification)	ThermoFisher Scientific	Cat#A1049101
ScaI-HF	NEB	Cat#R3122L
Streptavidin Alexa Fluor 647 conjugate	ThermoFisher Scientific	Cat#S21374
Triton X-100 solution	Sigma-Aldrich	Cat#93443
TrypLE Express Enzyme (1X), no phenol red	ThermoFisher Scientific	Cat#12604013
Xanthine	Sigma-Aldrich	Cat#X7375
**Critical commercial assays**		
Arginase Activity Assay Kit (Colorimetric)	Abcam	Cat#ab180877
Chromium Next GEM Chip G Single Cell Kit, 16 rxns	10x Genomics	Cat#PN-1000127
Chromium Next GEM Single Cell 3' GEM, Library & Gel Bead Kit v3.1, 16 rxns	10x Genomics	Cat#PN-1000121
Chromium Single Cell 3' Feature Barcode Library Kit, 16 rxns	10x Genomics	Cat#PN-1000079
DNeasy Blood and Tissue Kit	Qiagen	Cat#69506
P3 Primary Cell 4D-Nucleofector™ X Kit S	Lonza	Cat#V4XP-3032
Pierce BCA Protein Assay Kit	ThermoFisher Scientific	Cat#23227
**Deposited data**		
Single-cell RNA-sequencing data	This paper	Gene Expression Omnibus: GSE229505
**Experimental models: Cell lines**		
Human foreskin fibroblast	ATCC	SCRC-1041
**Experimental models: Organisms/strains**		
Mus musculus C57BL/6J	Jackson Laboratory	Cat#000664; RRID:IMSR_JAX:000664
Mus musculus B6.Cg-Gt(ROSA)26Sortm6(CAG-ZsGreen1)Hze/J (Ai6 Cre reporter)	Jackson Laboratory	Cat#007906; RRID:IMSR_JAX:007906
Toxoplasma gondii RHΔHXGPRT	Donald et al.[71]	N/A
Toxoplasma gondii RHΔKU80	Huynh and Carruthers[72]	N/A

(*Continued on next page*)

Continued

REAGENT or RESOURCE	SOURCE	IDENTIFIER
Toxoplasma gondii RHΔKU80 GRA59-HA	This paper	N/A
Toxoplasma gondii RHΔKU80 SOS1-HA	This paper	N/A
Toxoplasma gondii RHΔMYR1	This paper	N/A
Toxoplasma gondii RHΔGRA59	This paper	N/A
Toxoplasma gondii RHΔGRA59::GRA59-HA	This paper	N/A
Toxoplasma gondii RHΔKU80::GRA16-HA	This paper	N/A
Toxoplasma gondii RHΔMYR1::GRA16-HA	This paper	N/A
Toxoplasma gondii RHΔGRA59::GRA16-HA	This paper	N/A
Toxoplasma gondii RHΔKU80::IST-HA	This paper	N/A
Toxoplasma gondii RHΔMYR1::IST-HA	This paper	N/A
Toxoplasma gondii RHΔGRA59::IST-HA	This paper	N/A
Toxoplasma gondii RHΔROP16	This paper	N/A
Toxoplasma gondii RHΔSOS1	This paper	N/A
Toxoplasma gondii RHΔSOS1::SOS1-HA	This paper	N/A
Toxoplasma gondii CEPΔHXGPRT	Laboratory of John Boothroyd	N/A
Toxoplasma gondii CEPΔROP16	Tuladhar et al.[65]	N/A
Toxoplasma gondii CEPΔSOS1	This paper	N/A
Oligonucleotides		
Refer to Table S10	This paper	N/A
Recombinant DNA		
Refer to Table S11 for plasmids generated in this paper	This paper	N/A
pCas9-GFP-T2A-HXGPRT::sgRNA(PacI-NcoI)	Young et al.[7]	N/A
pCas9-GFP::sgRNA(UPRT)	Shen et al.[38]	Addgene Plasmid #54467
pUPRT	Shen et al.[38]	Addgene Plasmid #58528
pProGRA1-mCherry-T2A-HXGPRT-TerGRA2	Young et al.[7]	N/A
pHA-TerGRA2::ProDHFR-HXGPRT-TerDHFR	Butterworth et al.[10]	N/A
pTKO2	Rosowski et al.[29]	N/A
Software and algorithms		
Cell Ranger v3.0.2	10x Genomics	10xgenomics.com
Cell Ranger v.6.1.2	10x Genomics	10xgenomics.com
Harmony	PerkinElmer	perkinelmer.com/uk/product/harmony-4-9-office-license-hh17000010
Hotelling v1.0-8	Curran and Hersh	CRAN.R-project.org/package=Hotelling
Image Studio v5.2	LI-COR Biosciences	licor.com/bio/image-studio/
ImageJ v1.53c	Schneider et al.[73]	imagej.net
R v4.3.1	R Core Team	r-project.org
RStudio 2023.06.0 Build 421	Posit	posit.co
Seurat v4.3.0.1	Hafemeister and Satija[74]; Choudhary and Satija[75]	satijalab.org/seurat
VISION v3.0.1	DeTomaso et al.[32]	github.com/YosefLab/VISION

RESOURCE AVAILABILITY

Lead contact

Further information and requests for reagents may be directed to and will be fulfilled by the lead contact, Moritz Treeck (moritz.treeck@crick.ac.uk; mtreeck@igc.pt).

Materials availability

All plasmids and *T. gondii* cell lines generated in this study are available upon request.

Data and code availability

Single cell RNA-sequencing data (demultiplexed FASTQ files and Cell Ranger count matrices) have been deposited at the Gene Expression Omnibus with the accession number GSE229505.

All original code and supporting files used to analyse the single-cell RNA-sequencing data in this paper are available on GitHub at https://github.com/simonwbutterworth/Dual-Perturb-Seq-2023/.

Any additional information required to reanalyse the data reported in this paper is available upon request.

EXPERIMENTAL MODEL AND SUBJECT DETAILS

Mice

Wild-type C57BL/6J mice were bred and housed in pathogen-free conditions at the Biological Research Facility of the Francis Crick Institute in accordance with the Home Office UK Animals (Scientific Procedures) Act 1986 and European Union Directive 2010/63/EU. No regulated procedures were carried out on live animals at the Francis Crick Institute in this work.

Ai6 Cre reporter mice[76] were bred and housed in specific-pathogen-free conditions at the University of Arizona Animal Care facilities with a 14 hr/10 hr light/dark cycle, with ambient temperature between 68° and 75° F, and 30–70% humidity. All procedures and experiments were carried out in accordance with the Public Health Service Policy on Human Care and Use of Laboratory Animals and approved by the University of Arizona's Institutional Animal Care and Use Committee (#12-391).

Cell culture

HFF

Human foreskin fibroblasts (ATCC, SCRC-1041) were cultured in Dulbecco's Modified Eagle's Medium with 4.5 g/L glucose and GlutaMAX (Gibco) supplemented with 10% heat-inactivated foetal bovine serum (Gibco) at 37 °C and 5% CO_2.

BMDM

Monocytes isolated from the bone marrow of 6-12-week-old male C57BL6/J mice were differentiated into macrophages for six days in 70% RPMI 1640 ATCC modification (Gibco), 20% L929 cell-conditioned medium (provided by the Cell Services Science Technology Platform at the Francis Crick Institute), 10% heat-inactivated FBS (Gibco), 100 U/mL penicillin-streptomycin (Gibco) and 50 μM 2-mercaptoethanol (Sigma). Following differentiation, 2-mercaptoethanol was removed from the medium.

Primary neurons

Primary murine neuronal cell cultures were derived from Ai6 Cre reporter mice as previously described.[77] Briefly, cortical neurons were dissected from E17 embryos, dissociated, and seeded in poly-L-lysine-coated 96-well, clear-bottom, black-walled plates at a density of 20,000 cells per well in Minimal Essential Medium (Thermo) supplemented with D-glucose, L-glutamine, and 5% FBS. After four hours, the medium was changed to Neurobasal Medium (Thermo) with B-27 Supplement (Thermo), L-glutamine, and penicillin-streptomycin. After four days of *in vitro* culture, neurons received a half-volume medium change with complete Neurobasal Medium additionally supplemented with 5 μM cytosine arabinoside to prevent glial proliferation. Thereafter, one third medium exchanges with complete Neurobasal Medium occurred every 3-4 days. Experiments were carried out using the neurons following 10-12 days of *in vitro* culture.

Toxoplasma gondii

T. gondii tachyzoites were maintained by serial passage in HFFs every 2-3 days and isolated for experiments by passing through a 27-gauge needle followed by a 5 μm filter. Parental strains used in this study were RHΔHXGPRT,[71] RHΔKU80,[72] and CEPΔHXGPRT.[65] Parasite genotype was verified by restriction fragment length polymorphism of the SAG3 gene as previously described.[78] All *T. gondii* strains were regularly checked for *Mycoplasma spp.* contamination by PCR.

METHOD DETAILS

Generation of perturb-seq vectors

Capture sequences 1 and 2 were inserted into the pCas9-sgRNA vector[7] by inverse PCR using primers 1-4 (Table S9). To generate individual perturb-seq vectors, the protospacer was modified by inverse PCR using primers 5-29 (Table S9). To generate the pool of perturb-seq vectors used in the screen, ssDNA oligonucleotides encoding the protospacer sequences were selected from an arrayed library[7] using an Echo 550 Acoustic Liquid Handler (Labcyte). Oligonucleotides were dispensed in triplicate on three different days to reduce loss due to incomplete thawing of frozen stocks or misalignment of the plates. The pooled oligonucleotides were integrated into the pCas9-sgRNA-CS1 vector by Gibson assembly as previously described.[7] An Illumina sequencing library was prepared from the resulting plasmid pool to verify incorporation of the protospacer sequences by nested PCR using primers 30-33 (Table S9). The resulting library was sequenced on a HiSeq 4000 platform (Illumina) with 100 bp paired-end reads to a depth of 30 million reads (Table S3). The numbers of reads matching each protospacer sequence were counted using a custom perl script.

Dual perturb-seq experiments

T. gondii RHΔHXGPRT were transfected with KpnI-HF-digested (NEB) perturb-seq vectors by electroporation using the Amaxa 4D Nucleofector system (Lonza) with buffer P3 and pulse code EO-115. For individual perturb-seq vectors, at least 10^7 parasites were transfected with 15 μg of plasmid in one well of a 4D Nucleofector X Unit 16-well strip. For the perturb-seq plasmid pool, at least 10^8

parasites were transfected with 120 μg of plasmid split across eight wells of a 16-well strip. 24 h after transfection, 25 μg/mL mycophenolic acid (Sigma) and 50 μg/mL xanthine (Sigma) were added to the parasites to select for integration of the linearized perturb-seq vector into the genome for at least seven days.

24 h prior to infection, the HFF medium was changed to Dulbecco's Modified Eagle's Medium with 4.5 g/L glucose and GlutaMAX supplemented with 2% heat-inactivated FBS. The HFFs were infected with transfected parasites at a MOI of 0.1. After 1 h the medium was changed to remove remaining extracellular parasites. Where IFNγ stimulation was used, recombinant human IFNγ (BioTechne) was added to the infected cells at 21 h post-infection to a final concentration of 5 ng/mL (~10 U/mL).

24 h post-infection, the HFFs were dissociated using TrypLE Express (Gibco) and stained with 5 μg/mL DAPI (Sigma) for 5 minutes on ice. The cells were pelleted by centrifugation at 300 x g for 5 minutes at 4 °C, resuspended in 2% FBS DMEM without phenol red, and passed through a 30 μm filter. Infected cells were enriched based on Cas9-GFP expression using a FACSAria III cell sorter (BD).

The sorted cells were concentrated by centrifugation at 300 x g for 5 minutes at 4 °C and resuspended in DPBS (Sigma) with 0.04% w/v BSA (Sigma). The cells were partitioned into gel bead-in-emulsion droplets for reverse transcription using the Chromium Controller (10x Genomics) and Single Cell 3' GEM, Library & Gel Bead Kit v3.1 (10x Genomics) according to the manufacturer's instructions, with a targeted recovery of 10,000 cells per channel. Reverse transcription within the gel bead-in-emulsion droplets incorporates a 16 bp cell barcode (unique to each droplet) and 12 bp unique molecular identifier (UMI, unique to each transcript) into each cDNA. 3' mRNA and CRISPR sgRNA sequencing libraries were constructed following the manufacturer's protocol for 3' gene expression with Feature Barcoding technology for CRISPR screening, using the additional Chromium Single Cell 3' Feature Barcode Library Kit. The libraries were sequenced on a HiSeq 4000 platform (Illumina) with a targeted depth of 50,000 reads per cell for the 3' mRNA library and 5000 reads per cell for the CRISPR sgRNA library.

Generation of *T. gondii* cell lines

C-terminal epitope tagging

Cas9-sgRNA plasmids (without HXGPRT)[38] targeting the 3' UTRs of GRA59 and SOS1 were generated by inverse PCR using primers 5, 34, and 35 (Tables S10 and S11). In-frame HA-Ter^{GRA2}::Pro^{DHFR-}HXGPRT-Ter^{DHFR} repair constructs were amplified from a plasmid template[10] using primers 36-39 to introduce 40 bp homology arms to the 3' UTRs of GRA59 and SOS1. For each strain, 15 μg each of Cas9-sgRNA plasmid and repair construct were co-transfected into the *T. gondii* RHΔKU80 by electroporation as described above. 24 h after transfection, 25 μg/mL mycophenolic acid (Sigma) and 50 μg/mL xanthine (Sigma) was added to the culture medium for at least seven days to select for integration of the repair construct into the genome, following which clonal cell lines were obtained by limiting dilution. Integration of the repair construct into the genome was verified by diagnostic PCR using primers 40-43 on parasite genomic DNA extracted using the DNeasy Blood and Tissue Kit (Qiagen).

Knockouts (T. gondii RH strain)

Cas9-sgRNA plasmids (without HXGPRT) targeting the coding sequence of the target gene were generated by inverse PCR using primers 5 and 44-47 (Tables S10 and S11). Pro^{GRA1}-mCherry-T2A-HXGPRT-Ter^{GRA2} repair constructs were amplified from a template plasmid using primers 48-55 to introduce 40 bp homology arms to the 5' and 3' UTRs of the target gene. For each target gene, 15 μg each of Cas9-sgRNA plasmid and repair construct were co-transfected into *T. gondii* RHΔKU80 by electroporation as described above. 24 h after transfection, 25 μg/mL mycophenolic acid (Sigma) and 50 μg/mL xanthine (Sigma) was added to the culture medium for at least seven days to select for integration of the repair construct into the genome, following which clonal cell lines were obtained by limiting dilution. Integration of the repair construct into the genome was verified by diagnostic PCR using primers 56-63.

Knockouts (T. gondii CEP strain)

A Cas9-sgRNA (without HXGPRT) with two sgRNAs targeting the 5' and 3' UTRs of SOS1 was generated as previously described[79] using primers 77-80 to introduce the protospacer sequences by inverse PCR (Tables S10 and S11). A repair plasmid to replace the coding sequence of SOS1 with an HXGPRT-mCherry construct was generated by amplifying the 5' and 3' UTRs of SOS1 (adjacent to the protospacers in the Cas9-sgRNA plasmid) using primers 81-84 and inserting the resulting fragments into the pTKO2 plasmid[29] by In-Fusion cloning (Takara). Parasites were co-transfected with both the Cas9-sgRNA and pTKO2 plasmids, selected with mycophenolic acid and xanthine, as clonal cell lines obtained as described above. Deletion of the SOS1 CDS was verified by diagnostic PCR on extracted genomic DNA using primers 85-86, with amplification of the SAG1 CDS with primers 87-88 used as a control. Loss SOS1 mRNA expression relative to actin mRNA was verified by quantitative real-time PCR using primers 89-92.

Complementation

The coding sequence and 1000-1500 bp upstream of the start codon of the target gene were amplified from RHΔKU80 genomic DNA using primers 64-71 (Tables S10 and S11) and integrated into the pUPRT plasmid[38] by Gibson assembly. 15 μg of pUPRT plasmid was linearised with AgeI-HF (NEB) (GRA59, SOS1, IST) or ScaI-HF (NEB) (GRA16) and co-transfected with 15 μg Cas9-sgRNA plasmid (without HXGPRT) targeting the UPRT locus. 24 h after transfection, 5 μM 5-fluorodeoxyuridine was added to the culture medium for at least seven days to select for integration of the pUPRT vector into the UPRT locus, following which clonal cell lines were obtained by limiting dilution. Integration of the pUPRT vector into the UPRT locus was verified by diagnostic PCR using primers 72-76.

Western blot

HA-tagged parasite cell lines

Parasites were isolated by syringe-lysis, filtering, and washing in DPBS then lysed in RIPA buffer (Thermo) supplemented with cOmplete Mini EDTA-free Protease Inhibitor Cocktail (Roche) and 2 U/mL benzonase nuclease (Sigma) for 30 min on ice. Protein concentration was quantified using the Pierce BCA protein assay kit (Thermo). The samples were centrifuged at 15,000 x g for 5 min at 4°C and the pellet discarded. 10 μg protein per sample was heated to 95 °C for 5 min in sample loading buffer and separated by SDS-PAGE using the Mini-PROTEAN electrophoresis system (Bio-Rad). Proteins were transferred to a nitrocellulose membrane using the Trans-Blot Turbo transfer system (Bio-Rad), blocked in 2% w/v skim milk powder, 0.1% v/v Tween 20 in PBS for 1 h at room temperature. The membrane was incubated in 1:1000 rat anti-HA (Sigma) or 1:200 mouse anti-*T. gondii* (Santa Cruz) for 1 h at room temperature followed by 1:10,000 goat anti-rat IRDye 800CW (LI-COR) or 1:10,000 goat anti-mouse IRDye 680LT (LI-COR) for 1 h at room temperature. Images were acquired using an Odyssey infra-red imaging system (LI-COR).

STAT6 phosphorylation in primary neurons

Primary neurons were infected for 24 h then protein was extracted by sonication in RIPA buffer supplemented with Protease Inhibitor Cocktail (Sigma) and Phosphatase Inhibitor Cocktail 2 (Sigma), as previously described.[80] 30 μg protein per sample was separated by SDS-PAGE, transferred to a PVDF membrane, and blocked as above. The membrane was stained with rabbit-anti STAT6 (Cell Signaling) or rabbit anti-phospho-STAT6 (Cell Signaling) plus mouse anti-β-actin (Cell Signaling). Blots were imaged using an Odyssey infra-red imaging system (LI-COR), total signal intensity quantified in Image Studio v5.2 (LI-COR), and the ratio of anti-phospho-STAT6 signal to anti-STAT6 signal calculated. Three biological replicates were carried out. Differences in the phospho-STAT6/STAT6 ratios between strains were tested by two-sided *t*-test with Bonferroni correction.

Plaque assay

100 parasites were inoculated onto a T25 flask of confluent HFFs and left undisturbed for 10 days. The flasks were stained with 0.5% w/v crystal violet (Sigma), 0.9% w/v ammonium oxalate (Sigma), 20% v/v methanol in distilled water for 15 min then washed with DPBS. To measure knockout efficiency, the number of plaques in flasks supplemented with 5 μM 5-fluorodeoxyuridine were counted relative to untreated flasks in three biological replicates. Differences in percentage knockout efficiency were tested by two-sided *t*-test with Bonferroni adjustment. To measure plaque size, images were taken using a GelDoc Go System (Bio-Rad) and plaque area quantified using ImageJ v1.53c.[73] Differences in plaque sizes between strains were tested by two-sided *t*-test with Bonferroni adjustment, using all measured plaques from one biological replicate.

Immunofluorescence assays

Localisation of GRA59 and SOS1

Confluent HFFs in 8-well chamber slides (Ibidi) were infected with *T. gondii* strains for 24 h then washed with PBS and fixed with 4% w/v formaldehyde (Sigma) for 15 min. The cells were permeabilized with 0.2% Triton X-100 (Sigma) for 15 min and blocked with 2% w/v BSA (Sigma) for 1 h. The cells were stained with 1:500 rat anti-HA (Sigma) followed by 1:1000 goat anti-rat Alexa 594 (Thermo). The cells were then stained with 1:200 mouse anti-*T. gondii* (Santa Cruz) or 1:1000 rabbit anti-*T. gondii* (Abcam) plus 1:1000 mouse anti-GRA1 (Dubremetz lab) or 1:1000 mouse anti-GRA2 (Dubremetz lab) or 1:1000 rabbit anti-GRA3 (Dubremetz lab) or 1:500 mouse anti-ROP1 (Abnova). Finally, the cells were strained with 5 μg/mL DAPI plus 1:1000 goat anti-mouse Alexa 647 (Thermo) and 1:1000 goat anti-rabbit Alexa 488 (Thermo), where the mouse anti-*T. gondii* primary antibody was used, or with 1:1000 goat anti-mouse Alexa 488 (Thermo) and 1:1000 goat anti-rabbit Alexa 647 (Thermo) where the rabbit anti-*T. gondii* primary antibody was used. All antibody incubations were carried out for 1 h at room temperature. Images were acquired on a Nikon Ti-E inverted widefield fluorescence microscope with a Nikon CFI APO TIRF 100x/1.49 objective and Hamamatsu C11440 ORCA Flash 4.0 camera running NIS Elements (Nikon).

c-Myc

HFFs were serum starved for 24 h (0.1% FBS), then infected for 24 h, fixed, permeabilized, and blocked as above. The cells were stained with 1:800 rabbit anti-cMyc (Cell Signaling) and 1:200 mouse anti-*T. gondii* (Santa Cruz) for 1 h, followed by 1:1000 goat anti-rabbit Alexa 488 (Thermo), 1:1000 goat anti-mouse Alexa 647 (Thermo), and 5 μg/mL DAPI for 1 h. For each sample, a 3x3 tiled image was acquired as above using a Nikon Plan APO 40x/0.95 objective. cMyc fluorescence intensity was quantified in ImageJ. DAPI signal was used to generate a mask for each host nucleus and the median cMyc intensity in each nucleus of infected cells was measured with the median background (non-nucleus) intensity subtracted. The median nuclear signal per sample was taken as representative of a replicate, and normalized by scaling to RHΔKU80 = 1 AU. Five biological replicates were carried out using HFFs prepared independently and infected on different days. Differences between strains were tested by two-sided Wilcoxon rank-sum test with Bonferroni correction.

EZH2

HFFs were infected for 24 h, fixed, permeabilized, and blocked as above. The cells were stained with 1:200 mouse anti-EZH2 (BD) and 1:1000 rabbit anti-*T. gondii* (Abcam) overnight at 4 °C, followed by 1:1000 goat anti-mouse Alexa 488 (Thermo), 1:1000 goat anti-rabbit Alexa 647 (Thermo), and 5 μg/mL DAPI for 1 h. Images were acquired and nuclear EZH2 fluorescence in infected cells was quantified as above for cMyc. Five biological replicates were carried out using HFFs prepared independently and infected on different days. Differences between strains were tested by two-sided Wilcoxon rank-sum test with Bonferroni correction.

Phospho-p38

HFFs were infected for 24 h, fixed, permeabilized, and blocked as above. The cells were stained with 1:800 rabbit anti-phospho-p38 (Cell Signaling) and 1:200 mouse anti-*T. gondii* (Santa Cruz) overnight at 4 °C, followed by 1:1000 goat anti-rabbit Alexa 488 (Thermo), 1:1000 goat anti-mouse Alexa 647 (Thermo), and 5 μg/mL DAPI for 1 h. Images were acquired and nuclear phospho-p38 fluorescence in infected cells was quantified as above for cMyc. Five biological replicates were carried out using HFFs prepared independently and infected on different days. Differences between strains were tested by two-sided Wilcoxon rank-sum test with Bonferroni correction.

IRF1

HFFs were infected and 50 ng/mL (~100 U/mL) recombinant human IFNγ added 1 hpi. The cells were fixed 24 hpi, permeabilized, and blocked as above. The cells were stained with 1:200 rabbit anti-IRF1 (Cell Signaling) and 1:200 mouse anti-*T. gondii* (Santa Cruz) overnight at 4 °C, followed by 1:1000 goat anti-rabbit Alexa 488 (Thermo), 1:1000 goat anti-mouse Alexa 647 (Thermo), and 5 μg/mL DAPI for 1 h. Images were acquired and nuclear IRF1 fluorescence in infected cells was quantified as above for cMyc, normalzsing to uninfected = 1 AU. Five biological replicates were carried out using HFFs prepared independently and infected on different days. Differences between strains were tested by two-sided Wilcoxon rank-sum test with Bonferroni correction.

Phospho-STAT6

HFFs were infected for either 1 h or for 24 h, with remaining extracellular parasites washed off at 1 hpi. The cells were fixed with 100% methanol for 15 min and blocked with 2% w/v BSA for 1 h. The cells were stained with 1:200 rabbit anti-phospho-STAT6 (Cell Signaling) and either 1:200 mouse anti-*T. gondii* (Santa Cruz) or 1:10,000 mouse anti-SAG1 (Laboratory of John Boothroyd) overnight at 4 °C, followed by 1:1000 goat anti-rabbit Alexa 488 (Thermo), 1:1000 goat anti-mouse Alexa 647 (Thermo), and 5 μg/mL DAPI for 1 h. Images were acquired and nuclear phospho-STAT6 fluorescence in infected cells was quantified as above for cMyc. Five biological replicates were carried out for each timepoint using HFFs prepared independently and infected on different days. Differences between strains were tested by two-sided Wilcoxon rank-sum test with Bonferroni correction.

GRA16-HA/IST-HA export

HFFs were infected for 24 h, fixed with formaldehyde, permeabilized, and blocked as above. The cells were stained with 1:500 rat anti-HA (Sigma), followed by 1:1000 goat anti-rat Alexa 594 (Thermo), followed by 1:1000 rabbit anti-*T. gondii* (Abcam), followed by 1:1000 goat anti-rabbit Alexa 647 (Thermo). For each sample, a 3x3 tiled image was acquired as above using a Nikon Plan APO 40x/0.95 objective. The proportion of exported GRA16-HA/IST-HA was quantified using ImageJ. DAPI signal was used to generate a mask for each host nucleus and anti-*T. gondii* signal was used to generate a mask for each vacuole. The median anti-HA signal in the background (non-nucleus and non-vacuole) was subtracted across the whole image, and the total anti-HA signal in the host nuclei and vacuoles was measured. Export ratio was calculated as the amount of GRA16-HA/IST-HA immunofluorescence signal in the host nuclei compared to that remaining in the vacuoles. To determine the percentage of vacuoles with IST-HA accumulations, the images were blinded and the vacuoles manually scored for the presence/absence of IST-HA accumulations. Five biological replicates were carried out using HFFs prepared independently and infected on different days. Differences between strains were tested by unpaired, two-sided *t*-test with Bonferroni correction.

Arginase activity assay

10^6 BMDMs per sample were infected with *T. gondii* parasites at a MOI of 1 for 36 h. Arginase activity in the cell lysate was measured using a colorimetric assay (Abcam). Two biological replicates were carried out with BMDMs prepared and infected on different days, with triplicate infections for each strain in each biological replicate. Differences between strains were tested by two-sided Wilcoxon rank-sum test with Bonferroni correction.

Identification of protein homologues

The AlphaFold structural prediction for SOS1 (TGGT1_222100) was accessed from the AlphaFold/EMBL-EBI database version 2022-11-01.[81,82] Homologues of SOS1 were identified using HMMER[83] with UniProtKB reference proteomes with an E-value cut-off of 0.01. No significant hits were found outside of Apicomplexa. Regions of >40% similarity (BLOSUM62) were identified by BLAST via VEuPathDB.[33] A phylogenetic tree of select homologues was generated by Clustal Omega.[84]

In vitro cyst formation assay

Cyst formation assays were carried out as previously described.[66] Briefly, primary neurons were cultured in 96-well, clear-bottom, black-walled plates and infected with indicated *T. gondii* strains at a MOI of 0.1. At 1, 2, and 3 days post-infection, the cells were fixed with 4% w/v formaldehyde for 15 min, then simultaneously permeabilized and blocked with 0.1% v/v Triton X-100 plus 3% w/v goat serum. The cells were stained with 1:10,000 rabbit anti-*T. gondii* (Thermo) and 1:500 biotinylated *Dolichos bisflorus* agglutinin (DBA) for 1 h, followed by 1:500 streptavidin Alexa 647 (Thermo), 1:500 goat anti-rabbit Alexa 488 and DAPI for 1 h. Wells were covered with 100 μLs of 1:50 Fluoromount-G for imaging. Entire wells were imaged at 20x magnification (69 fields of view per well) using an Operetta high content imaging system (PerkinElmer). Image analysis was carried out using Harmony (PerkinElmer). *T. gondii* vacuoles were identified by anti-*T. gondii* staining. Only vacuoles containing two or more parasites (defined by a minimum vacuole length of 8 μm and width of 4 μm) were considered for further analysis. The percentage of DBA-positive vacuoles was determined in each well, and the mean percentage across replicate wells for each strain taken to represent each biological replicate. Three biological replicates were carried out. Differences between strains were determined by two-sided *t*-test with Bonferroni correction.

QUANTIFICATION AND STATISTICAL ANALYSIS

Analysis of dual perturb-seq data

A schematic overview of the analysis can be found in Figure S1.

Pre-processing, filtering, and normalisation

A multiple species reference genome comprising *Homo sapiens* GRCh38 release 95 and *Toxoplasma gondii* ME49 TGA4 release 42 was generated using Cell Ranger v3.0.2 (10x Genomics). Using Cell Ranger v6.1.2 (10x Genomics), sequencing reads from the 3' mRNA and CRISPR sgRNA libraries were aligned to this reference and a custom reference of protospacer sequences, including intronic reads but leaving all other settings as the default. Cell Ranger aligns the reads to the transcriptome/protospacer sequence references, assigns reads to a cell barcode, and counts the number of UMIs for each gene/protospacer assigned to each cell barcode to generate a UMI x cell barcode count matrices (for further details see support.10xgenomics.com). All subsequent analyses were carried out in R v4.0.2 (www.r-project.org). UMI count matrices from Cell Ranger were imported into Seurat v4.2.0[85] with *H. sapiens* mRNA, *T. gondii* mRNA, and CRISPR sgRNA counts split into separate assays of a single Seurat object. Cell barcodes were filtered to retain only cells with a high total count of both *H. sapiens* and *T. gondii* mRNA UMIs, corresponding to infected cells. Cells with >10% UMIs derived from mitochondrial genome-encoded transcripts, characteristic of low-quality cells,[86] were removed. Finally, we filtered the cells to retain only those in which a single protospacer sequence was detected, with the aim of eliminating multiplet droplets or HFFs infected by multiple parasites. After filtering, fewer cells were recovered in the combined IFNγ-stimulated data compared to unstimulated (9,265 versus 15,920) due to apparently slightly reduced sorting accuracy (resulting in more uninfected cells being carried through), cell viability, and sgRNA transcript recovery. The filtered cells were classified based on the *T. gondii* gene target of the detected protospacer sequence. *H. sapiens* mRNA counts were normalized using SCTransform v2.[74,75] Replicates (Chromium channels) of the perturb-seq screen from both unstimulated and IFNγ-stimulated samples were merged and normalized using SCTransform v2, passing the replicate ID as a variable to regress out of the SCTransform residuals.

Identification of effector proteins

The SCTransform residuals ("scale.data" slot) of the top 3000 most variable genes were analyzed by principal component analysis using Seurat. For each *T. gondii* target gene, the distribution of infected cells in the top 20 principal components was compared to all other cells (excluding cells infected with known effector proteins) using Hotelling's t^2-test as implemented in the Hotelling v1.0.8 package. Hits were called based on a Benjamini-Hochberg-adjusted *p*-value of 0.01 or less.

Pseudo-bulk analyses

For each *T. gondii* target gene represented by at least 30 single cells, pseudo-bulk transcriptomes were generated from the mean SCTransform residuals of the top 3000 most variable genes. These pseudo-bulk profiles were analysed by principal component analysis. For effectors identified by Hotelling's t^2-test, Pearson correlation coefficients were calculated between the pseudo-bulk transcriptomes and clustered using the complete linkage method implemented in the hclust function of the stats package v4.0.4.

Identification of differentially expressed genes

In the pilot experiments, differentially expressed host genes were identified by two-sided Wilcoxon rank-sum test on the SCTransform-normalized data using the sg(UPRT)-expressing cells as a reference population. In the screen, differentially expressed host genes were identified for each effector by the same method using all other cells (excluding controls) as a reference population. *p*-values were adjusted using the Benjamini-Hochberg procedure.

Identification of differentially expressed pathways

Single cells were scored for the expression of gene sets in Pathway Interaction Database[45,46] using the SCTransform-normalized gene expression data with VISION v2.1.0.[32] VISION signature scores were converted to Z-scores and, for each *T. gondii* target gene represented by at least 30 single cells, up- or down-regulation of each pathway in cells infected with that knockout compared to all other knockouts (excluding known effector proteins) was tested by two-sided Wilcoxon rank-sum test. For the effector proteins identified by Hotelling's t^2-test, the mean pathway Z-score was plotted for each of the pathways differentially expressed by at least one effector (Benjamini-Hochberg-adjusted *p*-value ≤ 0.05). The effectors and gene sets were clustered using these mean Z-scores using the complete linkage method.

Comparison to published bulk phenotypes using VISION signature scores

Host cell genes differentially expressed upon infection with a *T. gondii* knockout strain compared to a wild-type strain were extracted from bulk RNA-seq/microarray data for seven effector proteins: MYR1 (6 hpi RHΔMYR1-infected HFFs),[16] HCE1 (6 hpi RHΔHCE1-infected HFFs),[17] IST (6 hpi RHΔIST-infected HFFs),[87] GRA16 (18-20 hpi RHΔGRA16-infected HFFs),[21] GRA24 (18-20 hpi RHΔGRA24-infected murine BMDMs),[22] GRA28 (RHΔGRA28-infected THP-1s, timepoint not stated),[23] and ROP16 (24 hpi type II::ROP16$^{\text{type I}}$ versus type II wild-type- infected HFFs).[25] Single cell transcriptomes were scored for the expression of these gene sets using the SCTransform-normalized gene expression data with VISION v2.1.0,[32] resulting in a "signature score" for each marker gene set for each cell. A higher signature score indicates that the same genes are up- or down-regulated in the single cell transcriptome data as in bulk transcriptome data. *T. gondii* knockout-infected cells were tested for higher scores of the corresponding signature compared to UPRT knockout (pilot experiments) or all other cells (screen experiment) by Wilcoxon rank-sum test.

Cell Host & Microbe, Volume *31*

Supplemental information

High-throughput identification of *Toxoplasma gondii* effector proteins that target host cell transcription

Simon Butterworth, Kristina Kordova, Sambamurthy Chandrasekaran, Kaitlin K. Thomas, Francesca Torelli, Eloise J. Lockyer, Amelia Edwards, Robert Goldstone, Anita A. Koshy, and Moritz Treeck

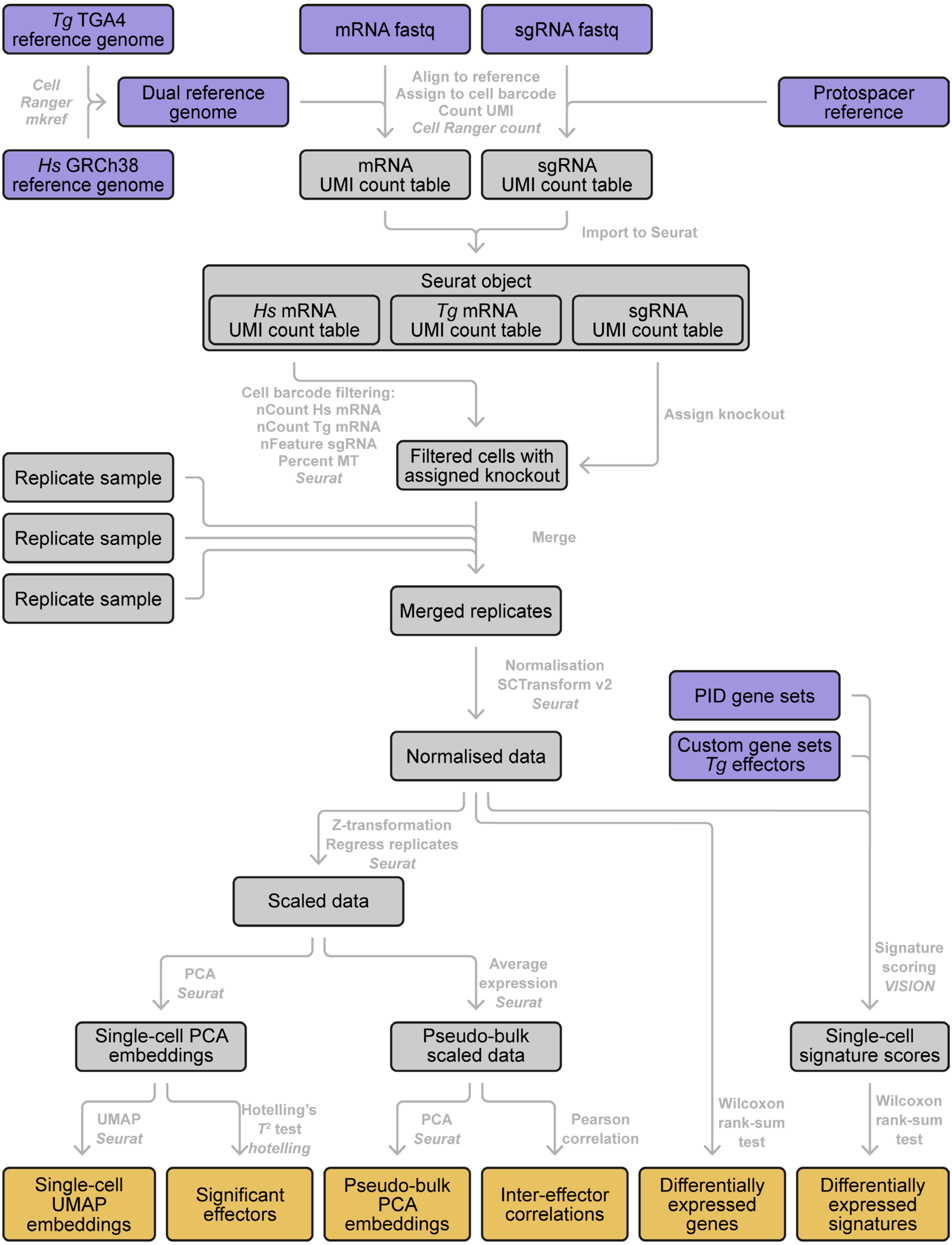

Figure S1. Schematic of dual perturb-seq data processing and analysis, related to STAR Methods.

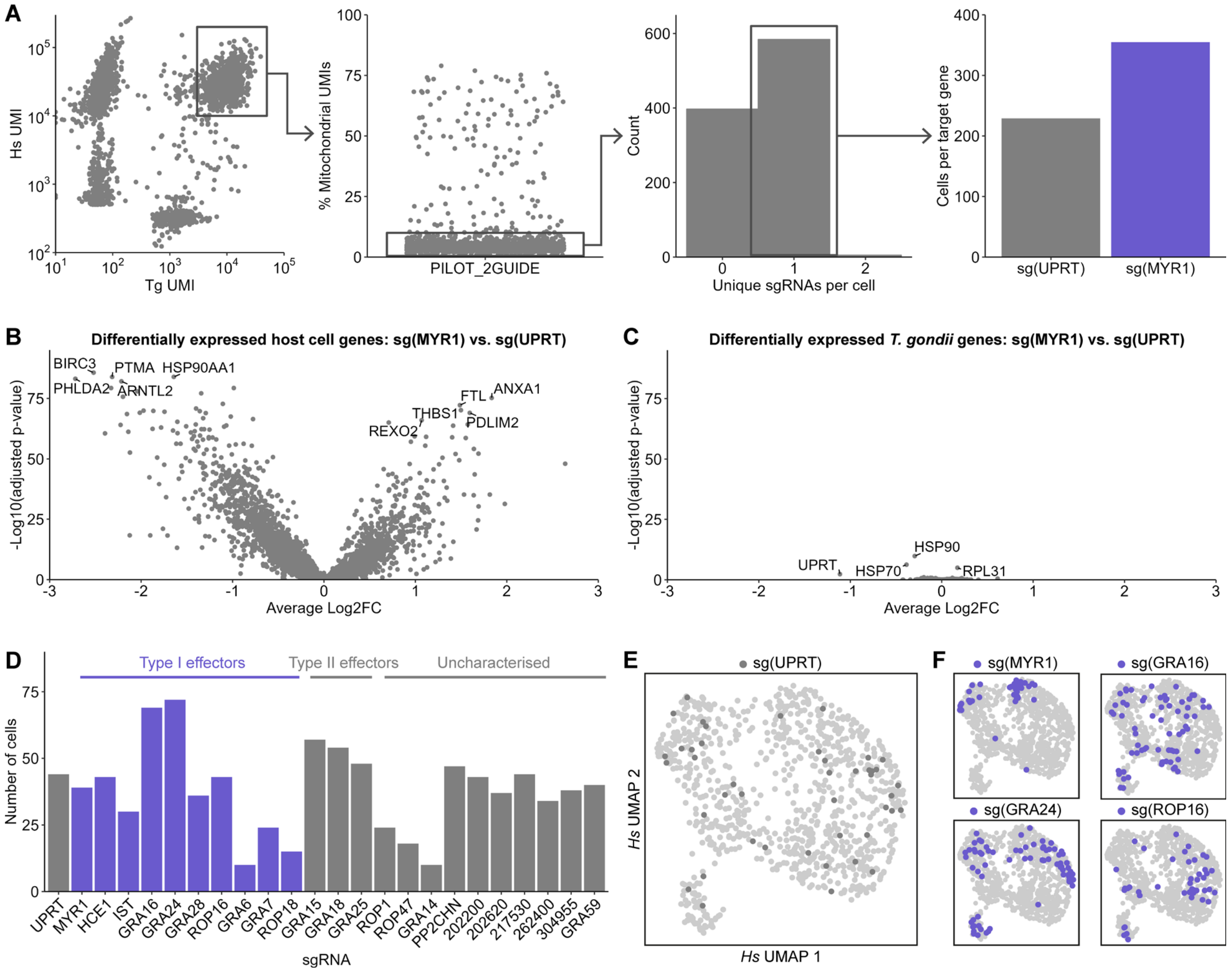

Figure S2. Filtering and assignment of sgRNA identity to dual perturb-seq transcriptomes, related to Figure 1.

A. Typical cell barcode filtering strategy for dual perturb-seq data. Cell barcodes are filtered to retain only those with high counts of both host cell and *T. gondii* UMIs, a low percentage of host cell UMIs deriving from mitochondrial transcripts, and in which only a single sgRNA species is detected.

B. Differentially expressed host cell genes for sg(MYR1)-expressing cells compared to sg(UPRT)-expressing cells in the 2-sgRNA pilot experiment (two-sided Wilcoxon rank-sum test with Benjamini-Hochberg adjustment). See also **Table S1A**.

C. Differentially expressed *T. gondii* genes for sg(MYR1)-expressing cells compared to sg(UPRT)-expressing cells in the 2-sgRNA pilot experiment (two-sided Wilcoxon rank-sum test with Benjamini-Hochberg adjustment). See also **Table S1B**.

D. Number of single cell transcriptomes recovered for each target gene in 24-sgRNA pilot experiment.

E. Distribution of sg(UPRT)-expressing cells in UMAP of host cell gene expression in 24-sgRNA pilot experiment.

F. Distribution of cells expressing sgRNAs targeting select effectors in UMAP of host cell gene expression in 24-sgRNA pilot experiment.

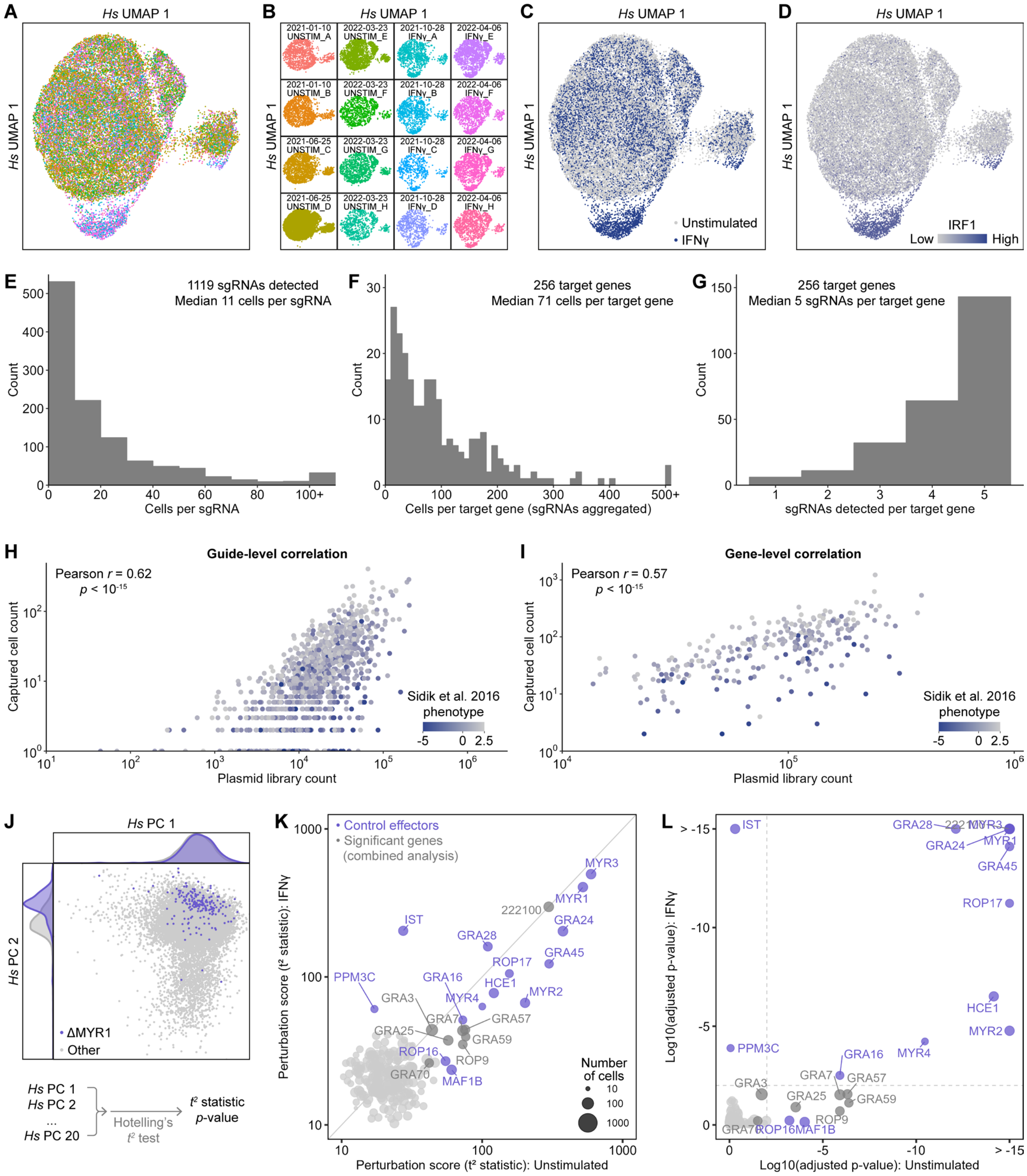

Figure S3. Quality control analysis of dual perturb-seq screen, related to Figure 2.

A. UMAP of single cell transcriptomes based on host cell gene expression coloured by sample.

B. UMAP of single cell transcriptomes split by sample.

C. UMAP of single cell transcriptomes coloured by condition (unstimulated or stimulated with IFNγ).

D. Expression of the interferon-stimulated gene IRF1.

E. Histogram of the number of single cell transcriptomes expressing each sgRNA. See also **Table S3**.

F. Histogram of the number of single cell transcriptomes for each target gene.

G. Histogram of the number of sgRNAs detected for each target gene.

H. Correlation between the number of read counts in bulk sequencing data of perturb-seq plasmid library and the number of single cell transcriptomes for each sgRNA. See also **Table S3**.

I. Correlation between the number of read counts in bulk sequencing data of perturb-seq plasmid library and the number of single cell transcriptomes summed for each target gene.

J. Illustration of Hotelling's t^2-test on single cell PCA embeddings.

K. Correlation between perturbation scores (Hotelling's t^2-test statistic) in unstimulated and IFNγ-stimulated samples. See also **Table S4**.

L. Correlation between Hotelling's t^2-test p-values in unstimulated and IFNγ-stimulated samples. See also **Table S4**.

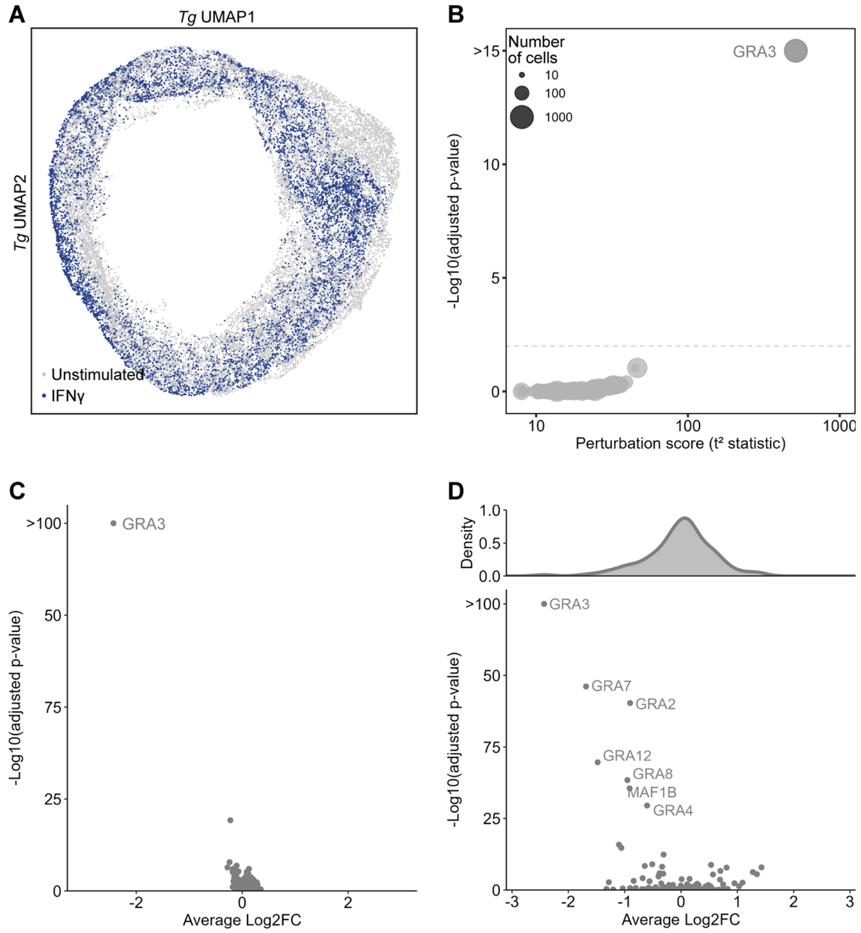

Figure S4. Perturbation of the *T. gondii* transcriptome by effector proteins, related to Figure 2.

A. UMAP of single cell transcriptomes based on *T. gondii* gene expression with cells coloured by condition (unstimulated or stimulated with IFNγ).

B. Perturbation of *T. gondii* transcriptome by effectors, measured by Hotelling's t^2 test on PCA embeddings of single cell transcriptomes with Benjamini Hochberg adjustment. See also **Table S5**.

C. Differentially expressed *T. gondii* genes for sg(GRA3)-expressing cells compared to all other cells (two-sided Wilcoxon rank-sum test with Benjamini-Hochberg adjustment). See also **Table S6**.

D. Differential expression of *T. gondii* genes targeted in this screen in cells expressing the cognate sgRNA that were detectably expressed in at least 25% of non-perturbed cells, with smoothed density estimate of average Log2FCs. See also **Table S7**.

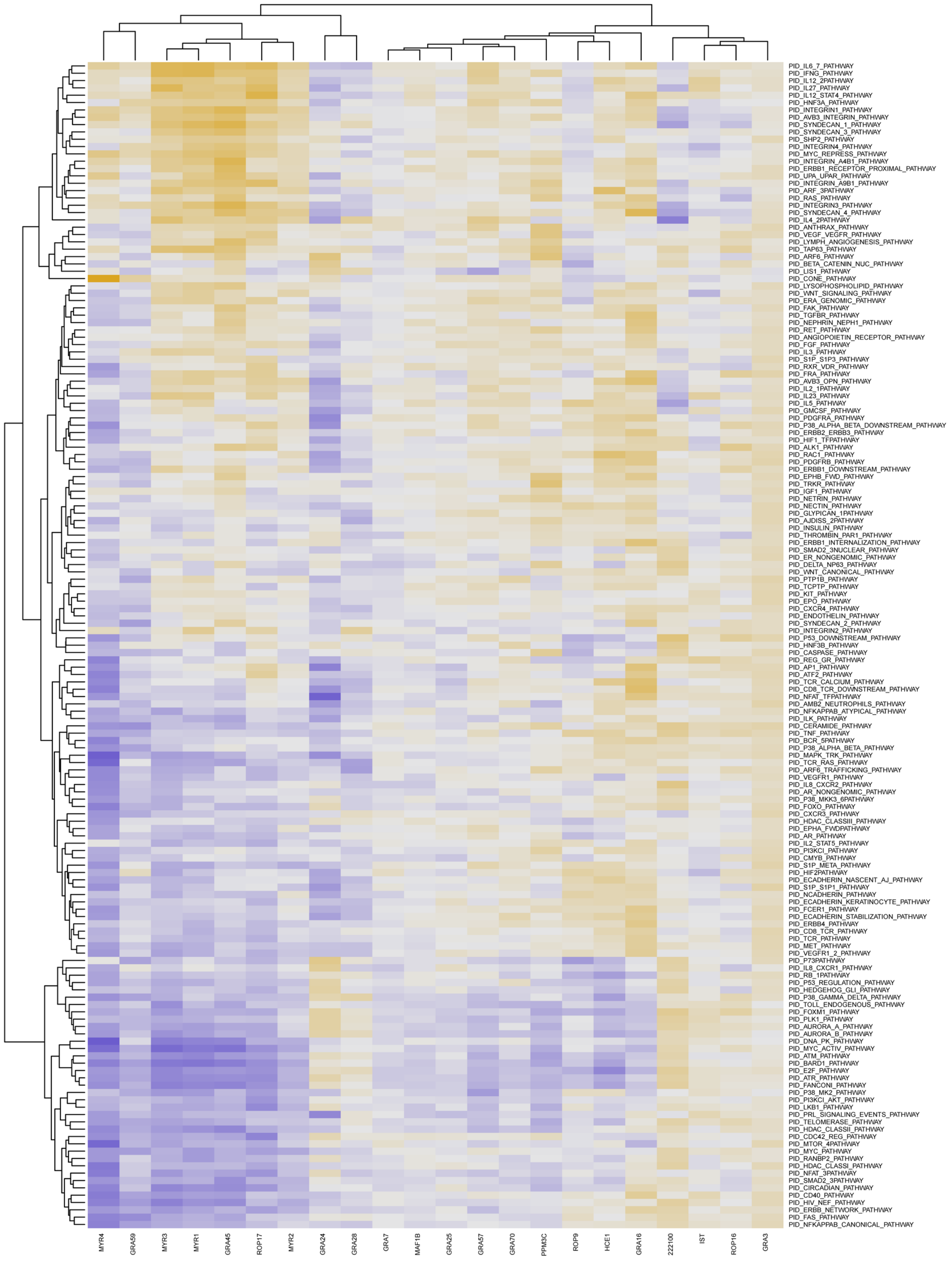

Figure S5. Pathway Interaction Database gene sets that are significantly differentially regulated by *T. gondii* effector proteins, related to Figure 2.

Average VISION signature scores of Pathway Interaction Database gene sets that are significantly differentially regulated by at least one significant effector ($p < 0.01$, two-sided Wilcoxon rank-sum test with Benjamini-Hochberg adjustment). See also **Table S8**.

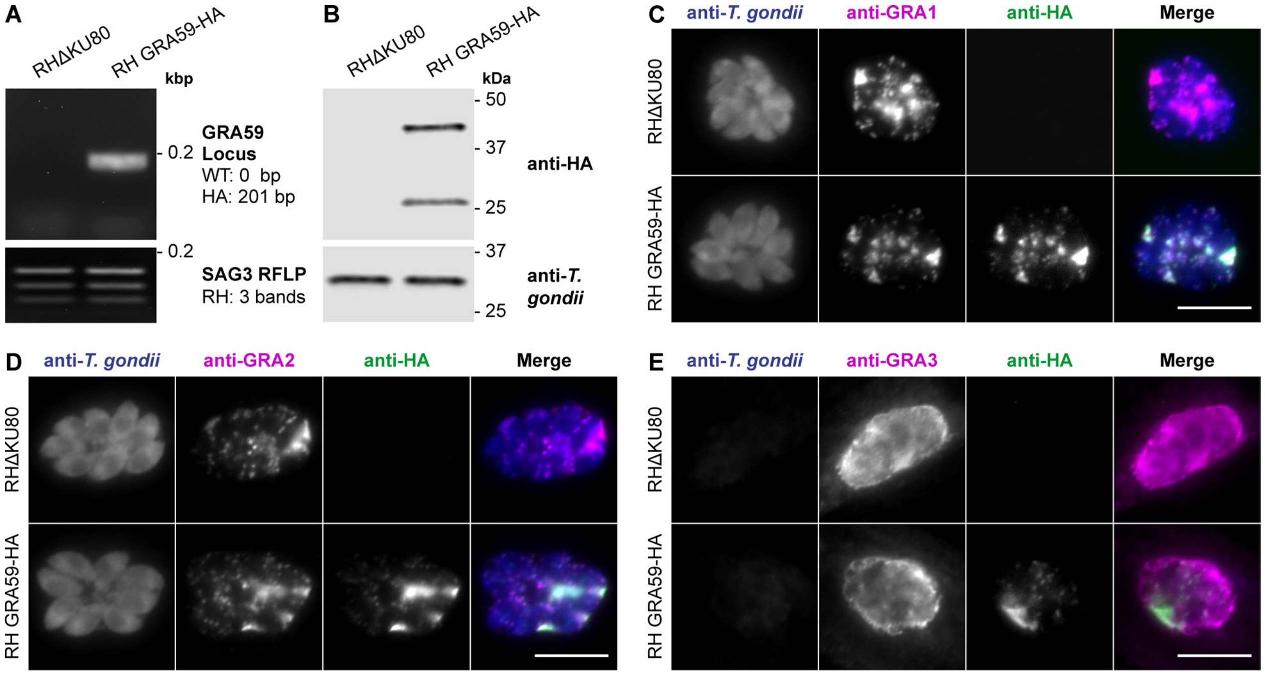

Figure S6. C-terminal epitope tagging of GRA59, related to Figure 3.

A. Verification of HA tagging by diagnostic PCR.

B. Verification of GRA59-HA expression by Western blot.

C. Co-localisation of GRA59-HA with GRA1 by immunofluorescence assay. Scale bar = 10 μm.

D. Co-localisation of GRA59-HA with GRA2 by immunofluorescence assay. Scale bar = 10 μm.

E. Co-localisation of GRA59-HA with GRA3 by immunofluorescence assay in cells permeabilised with 0.1% saponin for 15 min. Scale bar = 10 μm.

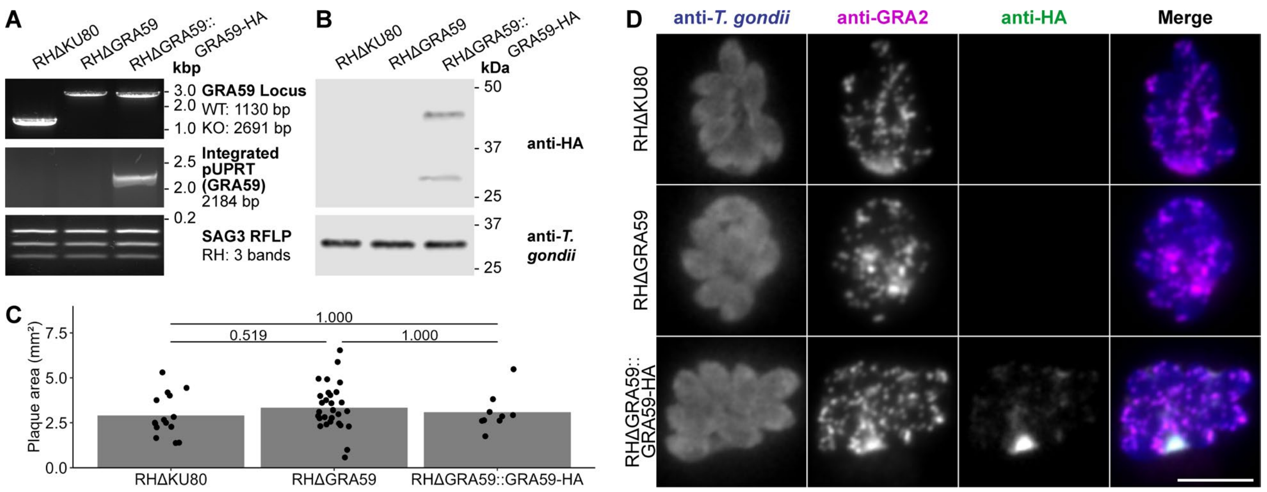

Figure S7. Knockout and complementation of GRA59, related to Figure 3.

A. Verification of GRA59 knockout and complementation by diagnostic PCR.

B. Verification of GRA59-HA expression by Western blot.

C. Plaque assay for RHΔKU80, RHΔGRA59, and RHΔGRA59::GRA59-HA. One biological replicate; points represent individually measured plaques. Differences tested by two-sided *t*-test with Bonferroni correction.

D. Verification of GRA59-HA expression and localisation by immunofluorescence assay. Scale bar = 10 μm.

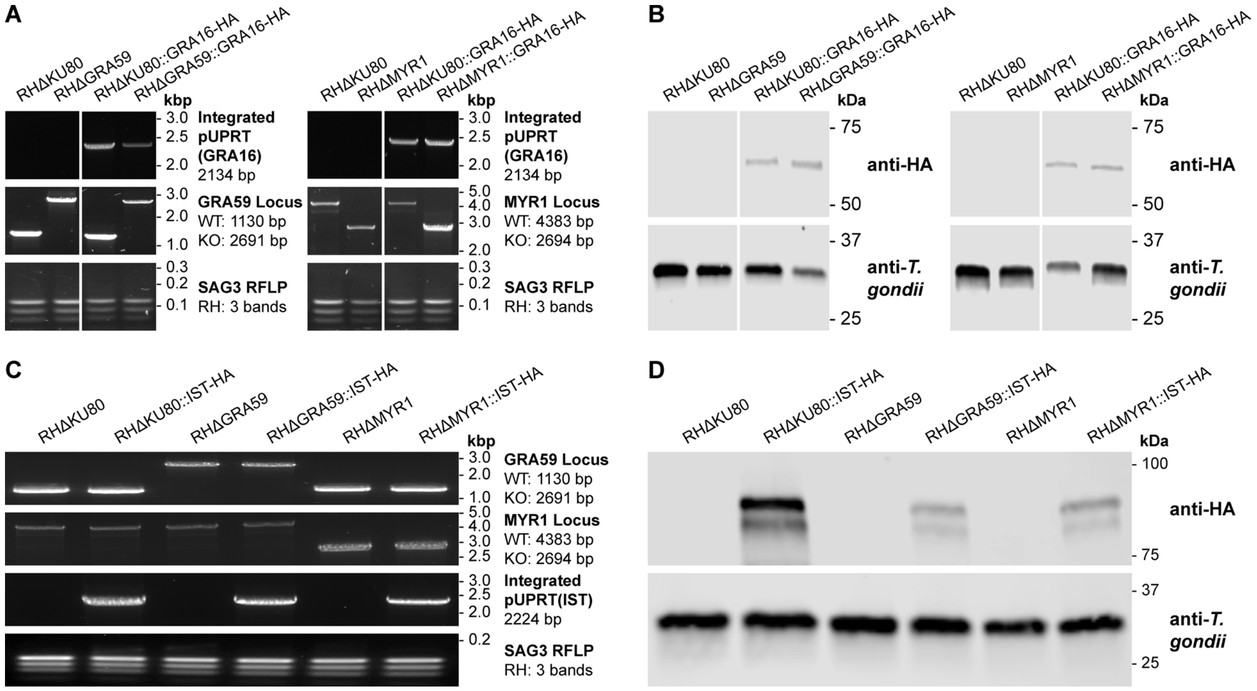

Figure S8. Introduction of GRA16-HA to GRA59 and MYR1 knockout cell lines, related to Figure 3.

A. Verification of pUPRT(GRA16-HA) integration by diagnostic PCR.

B. Verification of GRA16-HA expression by Western blot.

C. Verification of pUPRT(IST-HA) integration by diagnostic PCR.

D. Verification of IST-HA expression by Western blot.

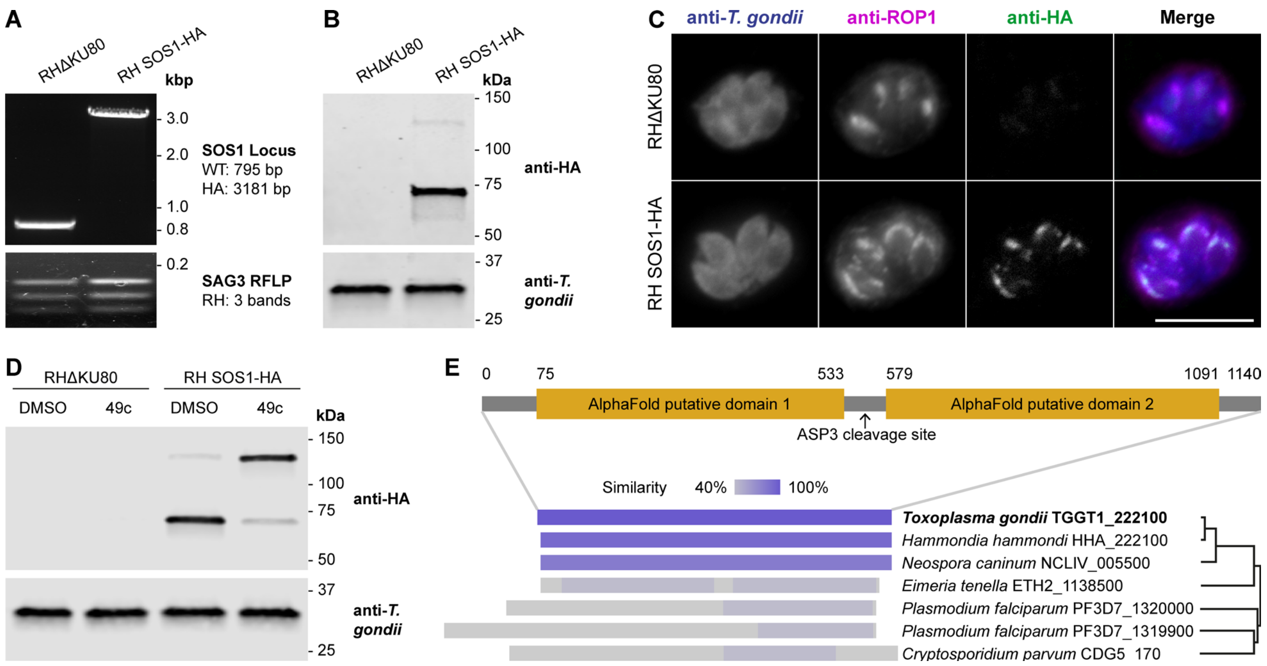

Figure S9. C-terminal epitope tagging of SOS1, related to Figure 4.

A. Verification of HA tagging by diagnostic PCR.

B. Verification of SOS1-HA expression by Western blot.

C. Co-localisation of SOS1-HA with ROP1 by immunofluorescence assay. Scale bar = 10 µm.

D. Treatment of parasites with the ASP3 inhibitor 49c reduces processing of SOS1. 10 µM 49c was added at 1 hpi and parasites were harvested by syringe-lysis at 48 hpi.

E. Putative structure of SOS1 and alignment to homologues detected in Apicomplexa.

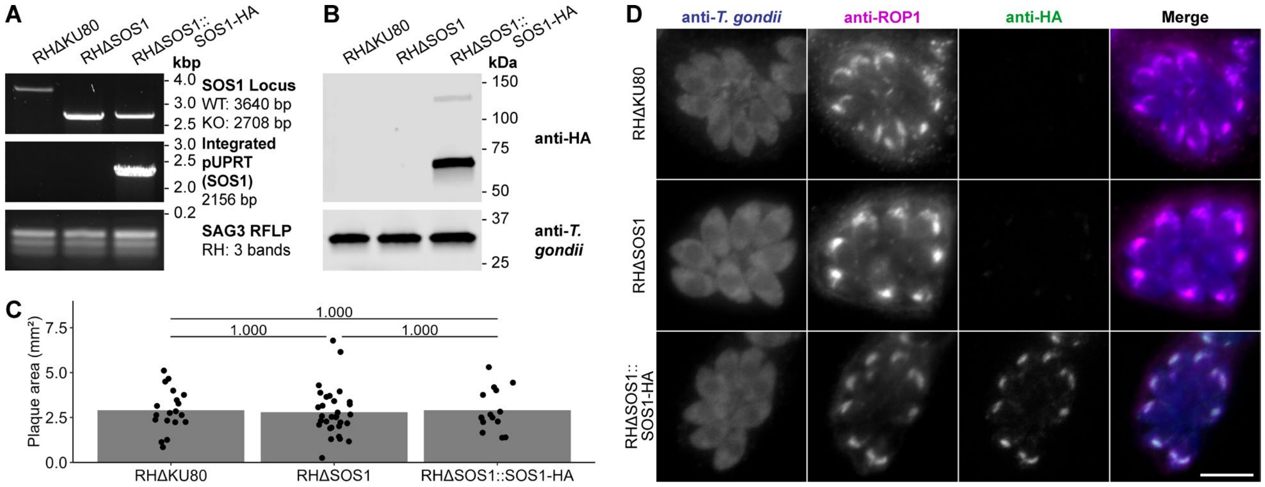

Figure S10. Knockout and complementation of SOS1 in *T. gondii* RH, related to Figure 4.

A. Verification of SOS1 knockout and complementation by diagnostic PCR.

B. Verification of SOS1-HA expression by Western blot.

C. Plaque assay for RHΔKU80, RHΔSOS1, and RHΔSOS1::SOS1-HA. One biological replicate; points represent individually measured plaques. Differences tested by two-sided *t*-test with Bonferroni correction.

D. Verification of SOS1-HA expression by immunofluorescence assay. Scale bar = 10 µm.

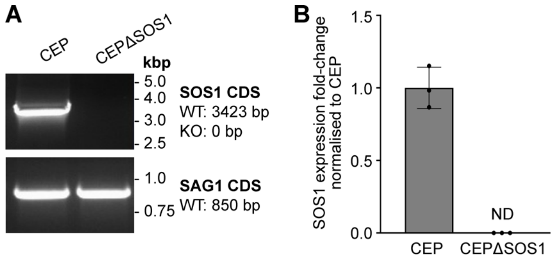

Figure S11. Knockout and complementation of SOS1 in *T. gondii* CEP, related to Figure 5.

A. Verification of SOS1 knockout by diagnostic PCR.

B. qPCR quantification of SOS1 mRNA expression relative to *Tg*Actin.
